# The driving mechanisms of the carbon cycle perturbations in the late Pliensbachian (Early Jurassic)

**DOI:** 10.1038/s41598-019-54593-1

**Published:** 2019-12-05

**Authors:** Luis F. De Lena, David Taylor, Jean Guex, Annachiara Bartolini, Thierry Adatte, David van Acken, Jorge E. Spangenberg, Elias Samankassou, Torsten Vennemann, Urs Schaltegger

**Affiliations:** 1 Department of Earth Sciences, University of Geneva, Geneva, Switzerland; 2 Department of Earth Sciences, University of Oregon, Portland, OR USA; 3 Department of Earth Sciences, University of Oregon, Eugene, OR USA; 4 Faculty of Geosciences and Environmental Sciences, University of Lausanne, Lausanne, Switzerland; 5 Center for Research on Palaeontology – Paris, UMR7207, MNHN, CNRS, SU, Paris, France; 6 Institute of Earth Sciences, University of Lausanne, Géopolis, Lausanne, Switzerland; 7 Irish Centre for Research in Applied Geosciences (iCRAG), UCD School of Earth Sciences, University College Dublin, Dublin, Ireland

**Keywords:** Biogeochemistry, Carbon cycle, Planetary science

## Abstract

The Early Jurassic (late Pliensbachian to early Toarcian) was a period marked by extinctions, climate fluctuations, and oceanic anoxia. Although the causes of the early Toarcian Oceanic Anoxia Event (OAE) have been fairly well studied, the events that lead to the Toarcian OAE, i.e. the events in the late Pliensbachian, have not been well constrained. Scenarios of the driving mechanism of biotic and environmental changes of the late Pliensbachian have ranged from LIP volcanism (the Karoo-Ferrar LIP), ocean stagnation, and changing ocean circulation, to orbital forcing. The temporal relationship between the Karoo LIP and the late Pliensbachian (Kunae-Carlottense ammonite Zones) are investigated in an effort to evaluate a causal relationship. We present the first absolute timescale on the Kunae and Carlottense Zones based on precise high-precision U-Pb geochronology, and additional geochemical proxies, for a range of environmental proxies such as bulk organic carbon isotope compositions, Hg concentration, and Hg/TOC ratios, and Re-Os isotopes to further explore their causal relationship. The data presented here show that causality between the Karoo LIP and the late Pliensbachian events cannot be maintained.

## Introduction

The Early Jurassic (Pliensbachian-Toarcian) was a period marked by recurrent biotic crises and environmental change. In the late Pliensbachian, there is compelling evidence that significant biotic and climatic changes took place^1–4^. For instance, a series of minor extinction events have been recorded in the Margaritatus Zone, at the Subnodosus, Gibbosus Subzones, and in the Spinatum Zone at the Hawskerense Subzone with extinctions happening at the species level (90–70%) in North-western European localities^2,5^. The same events have been reported in their correlative ammonite zones in North America, i.e., Kunae and Carlottense Zones, respectively^1,6^, highlighting the global extent of these extinction events. The carbon isotope record of the late Pliensbachian displays significant shifts, with a prominent positive shift below the Margaritatus-Spinatum boundary^3,7^ known as the late Pliensbachian event (LPE). This shift has been recognized in the carbonate carbon^4^, bulk organic carbon^3,8,9^, and terrestrial organic carbon^3,8^. Furthermore, important periods of production and preservation of organic matter (OM) take place in the late Pliensbachian, with potential evidence for an oceanic anoxic event (OAE)^10^. However, the causes of the extinction crisis and environmental changes are still conjectural. Because the events of the late Pliensbachian immediately precede the much larger early Toarcian OAE there has been speculation that the events of the late Pliensbachian might also be driven by the same magmatic event^5,6,11–13^. Due to the contrasting environmental conditions between the late Pliensbachian and the Toarcian OAE, the volcanic release of S-bearing aerosols has been evoked as the driving mechanism of the cool climatic conditions at this time^4,12,14^. However, definitive evidence for the occurrence of enhanced volcanism during the late Pliensbachian is still missing. Therefore, investigating the temporal relationship between the late Pliensbachian events and the Karoo-Ferrar LIP magmatism is crucial for potentially unravelling the causes of the climate fluctuations of this time. Lastly, the palaeoenvironmental changes of the late Pliensbachian have yet to be reported outside of the Western Tethys marine environments, which prevents a conclusive appreciation of the global effect of these environmental disturbances.

This paper aims to reconstruct the palaeoenvironmental changes of the late Pliensbachian outside of Western Tethys to evaluate the possible global extent of the climatic fluctuations and to test their temporal relation to LIP volcanism. To this aim, we have investigated the Nicely and Suplee Formations in the Izee-Suplee basin of the Blue Mountains terranes, East Oregon, USA^15,16^, an organic rich-mudstone series that spans the lower Kunae Zone to uppermost Carlottense Zone. High-precision U-Pb ages are presented to evaluate the temporal relation to LIP volcanism, in addition to bulk *δ*^13^C_org_, TOC, Rock Eval data, and Hg/TOC chemostratigraphy. Using these proxies for various environmental factors, we establish a precise timeline of environmental changes in the late Pliensbachian.

## Results

### Stratigraphic framework and ammonite biostratigraphy

**Figure 1:**
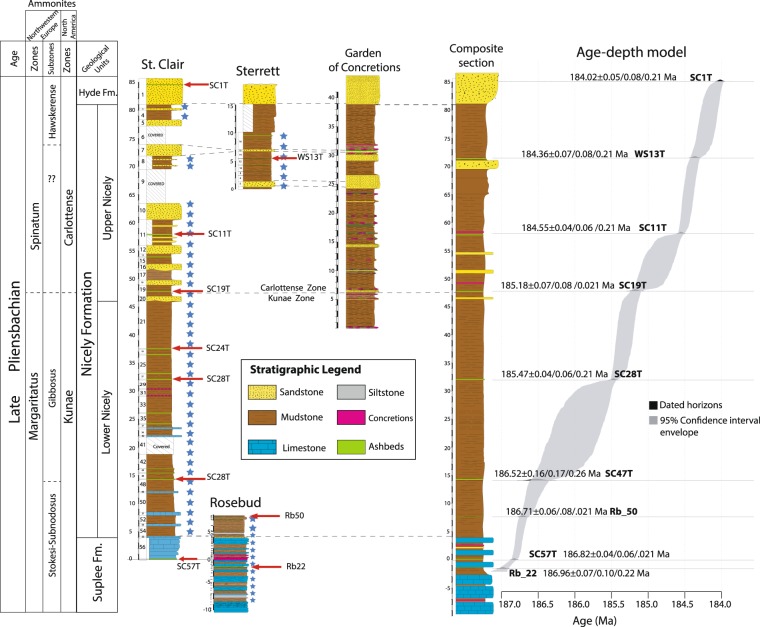
Stratigraphic sections and composite section in the Suplee, Nicely, and Hyde Formations (East Oregon, USA), showing ammonite biostratigraphy of the late Pliensbachian and correlations with NW European zonation. All sections were aligned with respect to the contact between stratigraphic units; the Rosebud and the St. Clair sections were aligned with respect to the contact between the Suplee and the Nicely Fm; the St. Clair, Sterrett, and Garden of Concretions with respect to the Hyde Fm. and Nicely Fm. contact. The sedimentation rate was assumed to be the same in all sections, thus a 1:1 relation of stratigraphic thickness between all sections was assumed. Blue stars represent the location of geochemical samples. All sampled points in each section were then projected onto the composite section. Each dated horizon corresponds to a U-Pb Th-corrected weighted mean (see FS. 2). All ages are reported as X/Y/Z where X includes analytical uncertainty only, Y includes analytical and tracer calibration uncertainty, Z includes analytical, tracer calibration and ^238^U decay constant uncertainty. The age-depth model envelope has 95% confidence interval. The age of the Kunae-Carlottense boundary is here dated at 185.18 ± 0.07 Ma.

Four sections were sampled: St. Clair, Sterrett, Rosebud, and Garden of Concretions (Fig. 1). These sections include the Suplee, and Nicely Formations and the base of the Hyde Fm., in the Izee terrane, OR, USA (FS. 1 Supplementary Information S.I). The stratigraphic thickness of the Suplee, Nicely and Hyde Fm sections are 14 m, 77 m, and 4 m, respectively. Ammonoids occur throughout the Suplee and Nicely Formations^17,18^ and encompass much of late Pliensbachian (Domerian). Two North American zones are recognized for that interval, the Kunae and Carlottense Zones (Fig. 1)^19^. Range charts giving the stratigraphic distribution of the ammonoids are provided in the SI.

### U-Pb geochronology and age-depth modelling

A total of ten ash beds were dated; SC1T = 184.02 ± 0.05/0.07/0.21 Ma, MSWD = 1.2, *n* = 7; WS13T = 184.36 ± 0.07/0.08/0.21 Ma, MSWD = 0.76, *n* = 4; SC11T = 184.55 ± 0.04/0.06/0.21 Ma, MSWD = 1.4, *n* = 5; SC19T = 185.18 ± 0.07/0.08/0.21 Ma, MSWD = 2.8, *n* = 3; SC24T = 183.92 ± 0.06/0.10/0.22, MSWD = 0.32, *n* = 5; SC28T = 185.47 ± 0.04/0.06/0.21 Ma, MSWD = 1.3, *n* = 4; SC_47T = 186.52 ± 0.16/0.17/0.26 Ma, MSWD = 0.53, *n* = 5; Rb_50 = 186.71 ± 0.06/0.08/0.21 Ma, MSWD = 1.2, *n* = 3, SC57T = 186.82 ± 0.04/0.06/0.21 Ma, MSWD = 0.52, *n* = 5, Rb_22 = 186.96 ± 0.07/0.10/0.22 Ma, MSWD = 0.67, *n* = 4 (FS. 2). Only one of the ten ash beds (SC24T 183.92 ± 0.06/0.10/0.22 Ma) yielding consistently younger U-Pb data that violated the stratigraphic superposition was excluded from the Bayesian age-depth model. We interpreted the date as being affected by Pb-loss. All Th-corrected ^206^Pb/^238^U ages are presented in FS. 2, and the raw data can be found in TS.1 (SI). The age-depth envelope with a 95% confidence interval can be found in Fig. 1 and age for every 10 cm of the stratigraphic record can be found in TS.2 (SI).

### Bulk organic carbon isotopes

**Figure 2:**
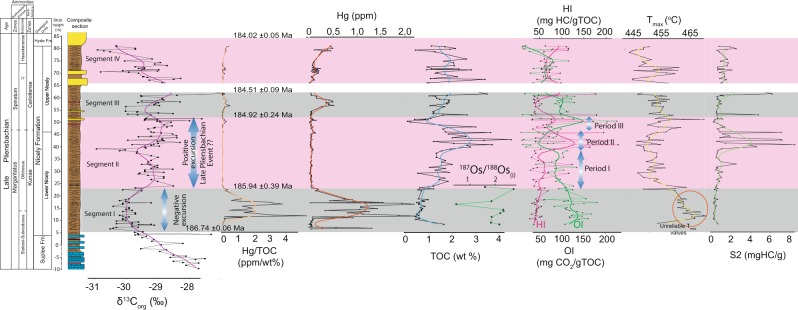
Geochemical data for the Suplee, Nicely, and Hyde Formations. Plotted proxies are, left to right, δ^13^C_org_, Hg/TOC (ppm/%), Hg (ppm), TOC (wt%), ^187^Os/^188^Os(i), HI (mg HC/gTOC), OI (mg CO_2_/gTOC), Tmax (°C), S2 (mg HC/g).

Three trends in the organic carbon isotope record have been recognized (Fig. 2, raw data in TS.3). In the lower Kunae Zone, the *δ*^13^C_org_ values decrease from −27.5 to −30.5‰ (Segment I), representing a −3.0‰ shift. In the middle-upper Kunae Zone, start a positive trend from −29.5 to −28.0‰. This shift lasts from the middle-upper Kunae Zone to the lower Carlottense Zone (Fig. 2; segment II). During the trend towards higher *δ*^13^C_org_ values, at the uppermost Kunae Zone, around the Kunae-Carlottense boundary, there is a short negative excursion to −31‰. After the positive trend, the *δ*^13^C_org_ values begin to become lower again; however, due to the 10-metre gap in the sedimentary record of the St. Clair, the trend is not so apparent although *δ*^13^C_org_ reach values of −30‰ (Fig. 2). The *δ*^13^C_org_ values from the Suplee Fm. and in the lower Nicely Fm. seem to display a fairly consistent trend. In the upper Nicely Fm., *δ*^13^C_org_ values are somewhat scattered.

### Re-Os isotope data

Samples from the lower Nicely Fm. show Re concentrations between 8.92 and 71.59 ppb, with the most Re-rich sample in the middle of the sequence (Fig. 2, data in TS.4). Osmium concentration varies to a lesser degree, between 240 and 552 ppt, resulting in ^187^Re/^188^Os ratios between 117 and 1737. Measured ^187^Os/^188^Os varies between 1.97 and 4.31 across the sequence. Initial ^187^Os/^188^Os values at 186.5 Ma are around 2 for the majority of samples, with some outliers to both higher and lower values (Fig. 2). One sample yields negative initial ^187^Os/^188^Os, suggesting the addition of Re. While concentrations for both Os and Re are comparable to values observed in other black shale suites in the late Sinemurian-early Pliensbachian (Robin Hood’s Bay, Yorkshire, UK^20^), the calculated initial ^187^Os/^188^Os values are notably more radiogenic (^187^Os/^188^Os of ~2 vs. 0.2–0.4).

### Rock Eval data

The variation of Rock Eval data with stratigraphic depth is plotted in Fig. 2, and other plots in FS. 4 and FS. 5 (data in TS.3). In the lower to middle Nicely Fm., TOC values increase steadily from 0.5% to 2.0%. Likewise, HI ranges from 27 to 50 mg HC/g TOC. The OI values show an inverse correlation with HI values and decrease from 150 to 60 mg CO_2_/g TOC. Around the Carlottense-Kunae boundary (~40 m of stratigraphic height) a sharp increase in TOC values reaches 4%, with a sharp increase in HI values to 50–228 mg HC/g TOC and a S2 peak reaching values > 4 mg HC/g, and a sharp decrease in OI values reaching 50 mg CO_2_/g TOC. Subsequently, TOC decreases to values close to 0% and so do HI values at 50–60 mg HC/g TOC. S2 peaks (the amount of hydrocarbons associated with kerogen) shift to values below 2 mg HC/g, and OI values increase, reaching values of 150–200 mg CO_2_/g TOC. Total organic carbon values increase again to close to 2%, HI values continue low ca. 50 mg HC/g TOC, S2 peaks cluster ca. 1 mg HC/g, OI values cluster ca. 100 mgCO_2_/gTOC. In the upper Nicely Fm., TOC values are scattered varying from 1 to 3%. Both HI and OI values are low clustering around 50–80 mg HC/g TOC and 50–80 mg CO_2_/g TOC, respectively. T_max_ values are high in the lowermost Nicely Fm. with values between 460–470 °C. For the middle to upper Nicely Fm. T_max_ values stay within the 444–460 °C range with some minor exceptions.

### Mercury data

Mercury concentrations were measured on selected samples (Fig. 2, data in TS.3). Mercury was normalized with TOC values from Rock Eval pyrolysis. An anomaly in Hg concentration is found at the lower Kunae Zone, with values reaching over 2 ppm. This spike in Hg concentration shows a positive covariance between the spike and the Hg/TOC values reaching beyond 2 (ppm/%). From the middle-upper Kunae Zone to the lower Carlottense Zone Hg and Hg/TOC values are considered background levels (Fig. 2). The second spike in Hg concentration is in the middle of the Carlottense Zone, with Hg concentrations reaching 0.5 ppm. The spike is not as prominent as that of the first spike; however, a positive covariance with Hg/TOC ratios is also observed with ratios reaching 0.25 (ppm/%).

## Discussion

### The global carbon isotope record of the late Pliensbachian

**Figure 3:**
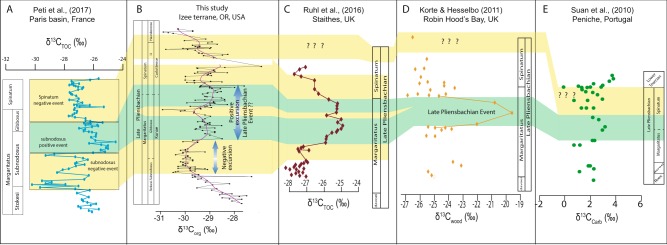
Global correlation of the carbon isotopic record of the late Pliensbachian. (**A**) Organic carbon isotope curve of^21^ for the Margaritatus and Spinatum Zones only; (**B**) Organic carbon isotope curve from Nicely, Suplee Formations, Oregon, USA, this study. The curve is the result of a moving average spline-fitting model; **(C**) Organic carbon data of the late Pliensbachian from the Staithes section, UK^8^; (**D**) Organic carbon isotope curve from the Robin Hood’s Bay, UK^3^; (**E**) Carbonate carbon isotope curve of the late Pliensbachian from the Peniche section, Portugal^4^.

To interpret our *δ*^13^C_org_ curve, we compared it with four other published carbon isotope curves for the same time interval. Here we attempt to build a global understanding of the carbon isotope fluctuations for the late Pliensbachian to potentially validate the environmental changes of the interval on a global scale. The *δ*^13^C_org_ record of the Nicely and Suplee Formations display several trends through the Kunae and Carlottense Zones that are very similar to those of the Western Tethys sections. In the lower Kunae Zone, the *δ*^13^C_org_ shift from −27.5 to −30.5‰, represents a −3.0‰ change (Fig. 3). In Europe, this negative shift is observed in the lower-middle Margaritatus Zone in the Staithes section and in the Paris Basin^21^ in the lower Subnodosus Subzone.

In the middle-upper Kunae Zone values have a range between −28.5 to −29.5‰, following a positive trend until the lower Carlottense Zone. In the Robin Hood’s Bay locality^3^, the shift occurs in the upper of the Margaritatus Zone at the Gibbosus-Subnodosus boundary, where it was named the “Late Pliensbachian event”^3^ (LPE), and is also reproduced in the Staithes locality^8^. The excursion is not reproduced in the Paris Basin^21^ at the same ammonite level. This location does, however, show a positive excursion at the upper Subnodosus Subzone and into the lower Gibbosus Subzone, which we interpret to be equivalent and attribute the small mismatch to possibly the resolution of ammonite data available. In the Peniche section^4^, Portugal, we suggest that the positive excursion spanning the upper Margaritatus and lower Spinatum would be correlative to both positive excursions at the same age as here. During this positive trend, at the uppermost Kunae Zone, just before the Kunae-Carlottense boundary, there is a short negative excursion; with *δ*^13^C_org_ values that reach −30 to −31‰. We tentatively interpret this excursion to be correlated to a small shift in the carbon isotope record from the Staithes section^8^ (Fig. 3), however, limited to a single data point. Neither in the Paris basin nor the Peniche section is this short negative excursion recognized.

In the middle of the Carlottense Zone, the carbon isotope values become more negative, and we correlate this negative trend as the negative trend in the lower to middle Spinatum Zone^8^. In the Paris Basin, we correlate this negative trend to be equivalent to the negative trend in the middle-upper Spinatum Zone. In the Peniche section, the values are fairly scattered in the upper Spinatum Zone but show an overall negative trend in the values. In summary, we argue that our carbon isotope curve from the middle Kunae to the uppermost Carlottense Zone does show similarities with other carbon isotope curves from North-western European sections. Nevertheless, the global extent of these variations is still debatable since in carbon isotope curves from other localities^14,22^ these fluctuations are not observed in the late Pliensbachian. Although, the absence of such carbon isotope fluctuations might be related to localised effects or reveal the incompleteness of the stratigraphic record, at least for some of these localities.

### Palaeoenvironmental implications

Our chemostratigraphic data display several important changes throughout the Kunae and Carlottense Zones (Figs. 2 & 3). In the lower Kunae Zone, there is a negative −3.0‰ shift in *δ*^13^C_org_ (Segment I; Fig. 2), and a coeval anomaly in the Hg concentrations and Hg/TOC. High Hg contents in marine sediments have been used to link environmental change and biotic crises to LIP activity in at least four cases in the Phanerozoic^13,23–27^. Additionally, the highly radiogenic ^187^Os/^188^Os_(i)_ values during the negative organic carbon excursion (N-CIE) (Fig. 2) indicate increased weathering of the continental crust, which has been attributed to the occurrence of LIP activity^28,29^. The OM deposited during the N-CIE has high T_max_ values > 460 °C, suggesting that the OM is over-mature. This suggests that burial overprint could have affected our geochemical proxies in this interval (Segment I). In fact, the S2 peaks (<0.5 mgHC/g) and TOC values (<0.5 mgHC/g) are low, but this could indicate either diagenetic overprint of the HI values as well as poor preservation of OM, e.g. in well-oxygenated bottom waters^30,31^. Recognizing one from the other is often difficult. Despite that, the overall T_max_ values from the Nicely Fm. are usually above 440 °C even for parts of the Nicely Fm that have high TOC and S2 peaks, which would indicate that post-depositional processes have affected the OM to some extent (FS. 4 & FS. 5). For instance, the large Hg/TOC spikes might be biased due to the low TOC values as a result of diagenetic overprint. Consequently, the HI and OI are, at least during the N-CIE, not reliable to infer on the nature of the OM because the TOC is too low. However, the isotopic composition of bulk organic carbon is not affected by hydrocarbon maturation or even low-grade metamorphism^32,33^. Therefore, there is no evidence to suggest that the N-CIE shift observed in the lower Kunae, or our *δ*^13^C_org_ dataset as whole, is the product of alteration. In terms of Re-Os systematics, when compared to other Early Jurassic sediment suites, the lower Nicely Fm. samples are comparable to other reported organic-rich mudstones^28,34–36^ with the exception of their very radiogenic ^187^Os/^188^Os values. Hydrocarbon maturation does not affect the isotopic system^37^, and substantial loss of ~45–80% of Re through hydrocarbon migration would be required to achieve initial ^187^Os/^188^Os typical for Jurassic seawater; which is considered unlikely given the high Re and Os concentrations measured (TS. 4). Thus, the unusually radiogenic initial ^187^Os/^188^Os values are unlikely to be the result of secondary processes.

**Figure 4:**
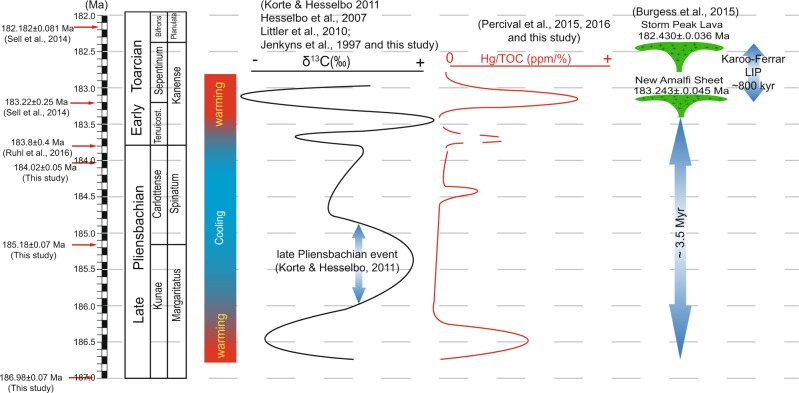
Temporal correlation between late Pliensbachian to the early Toarcian ammonite zones, carbon isotope variations, Hg/TOC anomalies, and the duration of the Karoo-Ferrar LIP. The duration of the Karoo-Ferrar LIP is based on high-precision U-Pb ages of^44^. The absolute timescale for the late Pliensbachian is based on ages from this study; those for the early Toarcian are based on^76^; the age of the Pliensbachian-Toarcian (Pl-To) boundary is astronomically calibrated from^8^. The δ^13^C curve for late Pliensbachian is an interpretation from^3,4,8,21^ and this study; the early Toarcian^3,9,77,78^. The Hg/TOC curve for the late Pliensbachian is based on the data from this study; Pl-To boundary to early Toarcian based on^13,29^. The Hg/TOC for the Pl-To boundary was left dashed since the spike is only present in one section^13^, thus not reproducible. Palaeoclimate variations of the late Pliensbachian to the early Toarcian are based on^3,4,67–70^.

Even though the link between the N-CIE and LIP is plausible geochemically, from a geochronological perspective, the connection seems problematic. The negative excursion is bracketed between 186.74 ± 0.06 Ma to 185.94 ± 0.39 Ma, deduced from our age model (Fig. 1). Our absolute timescale for the late Pliensbachian is compatible with a U-Pb high-precision age in the Kunae Zone at 185.49 ± 0.16 Ma in Canada^28^. A possible causal relationship between the Karoo-Ferrar LIP and the late Pliensbachian carbon cycle disturbance has been suggested based on the Ar-Ar chronometer^1,38^, and based on our temporal timescale for the late Pliensbachian the link is possible. However, biotic crises and environmental change in the Phanerozoic operate on a timescale that is significantly shorter than the analytical uncertainty produced by the Ar-Ar systematics. On average, the analytical precision of ^40^Ar/^39^Ar ages at 2σ is in the order of 1–2%, which for the Early Jurassic produces ages with ± 1.5–3.0 Ma resulting in an apparent long lifespan for the Karoo LIP of over 10 Myr^11^. Conversely, U-Pb high-precision geochronology has routinely produced ages at 0.1–0.05% precision. The use of a precisely calibrated EARTHTIME ^202^Pb-^205^Pb-^238^U-^235^U tracer solution^39,40^, improved error propagation algorithms^41,42^, the shift to single grains analysis, lowering of laboratory blanks, have all contributed to the successfulness of the technique^43^. As a result, high-precision U-Pb geochronology has shown that biotic crises and environmental change occur at 10^4^-10^5^ year timescale and constrains the lifespan of the most notorious Phanerozoic LIPs between 500–800 kyr^44–48^. Therefore, ^40^Ar/^39^Ar ages have not been successful in yielding sufficient temporal resolution to adequately assess causality between LIPs and biotic and environmental crises, and the link is often left elusive. This has been the case in at least all five major mass extinctions events in the Phanerozoic^49^. In the case of the N-CIE in the lower Kunae Zone, the excursion is 3.5 Myr older than the oldest known occurrence of the Karoo-Ferrar LIP, i.e., the New Amalfi Sheet at 183.243 ± 0.045 Ma^44^ (Fig. 4). Therefore, the lack of temporally coeval volcanic rocks of the Karoo-Ferrar LIP or other LIP to the N-CIE and Hg enrichment in the lower Kunae precludes any link to large scale volcanism as a driving mechanism.

Although the use of Hg/TOC as a geochemical proxy for LIP activity in the marine record has shown considerable promise in linking LIPs and some biotic crises and environmental change^13,23,25–27^, the link continues to be tenuous. The long-term enrichment of Hg in the marine environment is possible by other pathways than solely via oxidation of volcanic Hg^0^ delivered via rainfall. Volcanic Hg is readily absorbed and accumulated into the terrestrial reservoir (biomass and soils) and is a viable source of recycled Hg into the marine environment during environmental changes^24,50^. Furthermore, Hg/TOC as a geochemical proxy for LIP activity has only been successful in only a small number of cases. Percival *et al*.^51^ have shown that ^187^Os/^188^Os_(i)_ is much more consistently recording the effect of LIP volcanism than Hg/TOC in the marine record. Therefore, our geochemical evidence would indeed suggest LIP activity, although which LIP is still unknown. Oceanic hydrothermal activity input has been shown to enrich Hg in the marine record^52,53^; however, this scenario would be unlikely based on our highly radiogenic^187^Os/^188^Os_(i)_ data (Fig. 2).

Similar scenarios where marine Hg enrichment has no clear temporal connection to LIP activity are found in other cases in the Phanerozoic. In the Frasnian-Famennian biotic crisis, for instance, there is evidence for marine Hg enrichment^54^ and a global warming event^55^, but a temporal connection to any LIP activity is unclear and remains speculative. Moreover, astronomically forced climate change has proven successful in explaining the environmental change during the Frasnian-Famennian biotic crisis^56^. To explain the N-CIE and Hg/TOC spike in the lower Kunae Zone, a similar driving mechanism might be proposed. For instance, orbitally forced environmental change has been postulated to potentially destabilize the cryosphere, which can become an important source of isotopically light carbon to the atmosphere-ocean system^57^. The increased *p*CO_2_ would trigger global warming and enhanced continental run-off, which potentially erodes large areas of organic-rich sediments or wetlands^58^. These could have also been a viable source of isotopically light carbon to the atmosphere-ocean system^50^ as well as a source of remobilized terrestrial Hg^24,50^ and radiogenic Os^28,29,36^ to the marine environment. Furthermore, induced global warming could also trigger the dissociation of methane-hydrates from continental margin sediments, which could have also contributed as a source of isotopically light carbon^59^. This scenario would explain the geochemical evidence from the lower Kunae Zone without the need to evoke LIP volcanism.

Subsequent to the negative excursion in the lower Kunae Zone, our geochemical data include a positive trend in the *δ*^13^C_org_, here interpreted to be correlative to the LPE (Segment II; Fig. 2). Our geochemical data allows the trend towards higher *δ*^13^C_org_ values to be further subdivided into three distinct periods with respect to the nature of OM being deposited. The first period (185.94 ± 0.40 Ma to 185.35 ± 0.14 Ma) is characterised; by the deposition of terrestrial OM (type III) with TOC values increasing from 0.8–2.0%. Period II (185.35 ± 0.14 Ma to 185.18 ± 0.07 Ma) characterised; by a mixture of type II and type III OM with perhaps an increase of type II OM (HI values > 200 HC/g TOC; OI values < 50 mgCO_2_/TOC) (Fig. 2) with an increase of TOC occurring around the Kunae-Carlottense boundary. During the upper Kunae and lower Carlottense Zone, we have better confidence in characterising; the OM deposited because TOC values (2–4 wt%) and S2 peak values (1–8 mgHC/g) are higher (Fig. 2) although still low when compared, for instance, to Toarcian OAE black shales^31^. Period III (185.18 ± 0.07 Ma to 184.92 ± 0.24 Ma) is characterised; by the deposition of type III OM and TOC values of < 2%. Period III (185.18 ± 0.07 Ma to 184.92 ± 0.24 Ma) is characterised; by the deposition on type III OM and TOC values also of <2%.

Overall, the OM from the Nicely Fm. is predominantly type III, with a small part being type II (Period II Fig. 2 & FS. 4,A/B; FS. 5). Furthermore, our characterisation; of the OM from the Nicely Fm. as predominantly type III OM is in agreement with other studies that report Rock Eval pyrolysis data from the Spinatum and Margaritatus Zones from western Tethys^31,60–63^ (FS. 5). T_max_ values for LPE are higher than in North-western European sections, which suggest that the OM is mature to over-mature (FS. 4C/D; FS. 5) and confirms a degree of alteration has affected the OM in the Nicely Fm. Even though secondary processes have, to some degree, affected the HI in the Nicely Fm., hydrocarbon migration has potentially left a more refractory OM residue, i.e., type III OM. Therefore, it follows that the variations in the carbon-isotope record from the Nicely Fm. are not the result of the mixing of different OM end-members with different carbon isotopic composition, but rather are mainly a variation in the *δ*^13^C_org_ of terrestrial OM reservoir. Therefore, the fluctuations potentially reflect changes in the global carbon reservoir at the Earth’s surface. In Western Tethys sections, the LPE positive *δ*^13^C shift has been suggested to have affected the global carbon cycle^3,4,14,64,65^. The coeval deposition of dark shales with high TOC values just below the Margaritatus-Spinatum boundary (at the Gibbosus-Subnodosus boundary) in European sections (Fig. 3), and now in North America (around the Kunae-Carlottense boundary, ca. 185.2 Ma; Fig. 2), provides further evidence that the positive *δ*^13^C trend of the LPE is potentially global. The preservation and production of organic carbon in several late Pliensbachian marine sections suggest that a minor OAE possibly took place during the LPE^3,4,8,10^, or at least during period II (185.35 ± 0.14 Ma to 185.18 ± 0.07 Ma Ma) where potentially a higher proportion of type II in the bulk OM is preserved (Fig. 2). However, the apparent coeval accumulation of organic-rich rocks below the Margaritatus-Spinatum boundary does not necessarily imply widespread oceanic anoxia. Alternatively, enhanced productivity in surface waters, coupled with a high sedimentation rate could result in the deposition and preservation of OM without resulting in an OAE. Furthermore, evidence for the preservation of terrestrial OM over marine OM during the late Pliensbachian (FS. 5) certainly favours this scenario, i.e., oceanic anoxia was not as severe to the degree at which marine OM would be preserved globally. Therefore, the existence of an OAE during this time is still conjectural.

Many palaeoclimatic studies in the Early Jurassic predict low temperatures in the upper Margaritatus Zone and into the Spinatum Zone^3,4,66–70^ (Fig. 4) of which the causes have been suggested to be the result of the Karoo-Ferrar LIP emplacement^1,6,11^. Large scale volcanism is believed to lower global temperatures via the protracted volcanic degassing of SO_2_ and the rapid conversion to sulphate aerosols^71–73^. From our data set, no connection to LIP volcanism can thus be established. During the LPE, Hg and Hg/TOC return to background levels, and from a temporal perspective, a causal connection to the Karoo-Ferrar LIP would be unlikely (Fig. 4) as the event is still ca.1.5 Myr older than the Karoo-Ferrar LIP. In the absence of a clear connection to the occurrence of a LIP, the degassing of SO_2_ sourced from LIPs is unlikely to be the process responsible for the cool and dry climates of the LPE.

In the middle of the Carlottense Zone (Segment III, Fig. 2) *δ*^13^C_org_ values show a N-CIE starting at 184.92 ± 0.24 Ma to 184.51 ± 0.09 Ma, with TOC values ranging from 1% to close to 2%, and the coincident increase in the Hg concentrations and Hg/TOC ratios. The Hg/TOC ratios range from 0.15 to 0.50 ppm/%, which is comparable to the Hg/TOC spikes during the Toarcian OAE^13^. Unfortunately, the Os isotopic composition of the Nicely Fm. was not obtained in this segment; therefore, a link to volcanism of the Karoo-Ferrar LIP is tenuous; additionally, from a geochronological perspective the link is also not substantiated (Fig. 4).

In Segment IV, the *δ*^13^C_org_ indicates a negative shift towards the Pliensbachian-Toarcian boundary, which appears to agree with the global carbon isotopic trend towards the Pliensbachian-Toarcian (Pl-To) boundary^13^. However, our geochemical record in the uppermost Pliensbachian is not continuous enough and does not allow any discussion on the events that take place at Pl-To boundary.

## Conclusions

The late Pliensbachian has been considered as a period of protracted cool and dry climate^3,4,70,74^. However, our detailed proxies for environmental factors support the hypothesis that pulsed and contrasting climatic conditions operated during the late Pliensbachian, with potential warm periods evident in the lower Margaritatus Zone and cooler climates around the Margaritatus-Spinatum boundary and into the Spinatum Zone^10,14^. Our organic geochemical proxies for the LPE, for the first time outside of the Western Tethys realm, provide further evidence for the global deposition of organic-rich rocks below the Margaritatus-Spinatum boundary. However, conclusive evidence for an OAE during the LPE is still lacking. The driving mechanisms for these environmental changes in the late Pliensbachian have been speculated to be the result of volcanism^1,4,6,10–12^, ocean stagnation^74^, changing ocean circulation^70^, and orbital climate forcing^57^. Here we have shown that LIP volcanism is an unlikely candidate for the driving mechanism since no LIP is known to occur between ca. 187–184 Ma. A causal link to the Karoo-Ferrar LIP has been suggested based on ^40^Ar/^39^Ar ages^1,6,11^; however, the low accuracy and analytical precision of the methodology hinders a more precise connection. As a result, based on high-precision U-Pb geochronology, the degassing of volcanic S-species (SO_2_) inducing an ice-house climate^12,72,75^, for instance, is an unlikely driving mechanism of the cool and dry climate of late Pliensbachian. The Hg/TOC spikes throughout the late Pliensbachian adds to the growing body of evidence showing that prominent deviations of the marine Hg/TOC record alone cannot be readily used as a proxy for LIP activity and is better when combined with high-precision U-Pb geochronology to accurately and precisely evaluate temporal relationship to LIPs. Finally, it has long been speculated that the events of both the late Pliensbachian and the early Toarcian were biotic and climatic responses to the same underlying driving mechanism; i.e. the emplacement of the Karoo-Ferrar LIP. Each of their contrasting climate conditions have been thought as being regulated by different stages of volcanic degassing of the Karoo-Ferrar LIP (e.g. CO_2_ vs SO_2_)^4,6^. However, our geochronological data effectively decouples the driving mechanisms of climate change between the late Pliensbachian (Kunae-Carlottense Zones) and early Toarcian as being separate and unrelated.

## Methods

### Stratigraphic sampling and sample processing

In the field, sections and beds were measured using a metre; stick, and numbered consecutively. The outcrops were cleaned with a brush, altered/weathered samples removed with the aim of collecting fresh samples when possible. Samples for geochemistry were collected every 30–50 cm (on average) of all stratigraphic sections with the exception of the Garden of Concretions. Ash beds were collected, whenever present. Samples for geochemical analysis were powered using an agate mill. Ash beds were processed using a tungsten mill, zircons separated by gravitational floating methods in water, magnetic separation and heavy liquid density separation.

### U-Pb geochronology

The geochronological method of choice was U-Pb zircon CA-ID-TIMS because yields ^206^Pb/^238^U dates at 0.1–0.05% precision. The depositional age of ash beds was calculated from the weighted means of the youngest overlapping ^206^Pb/^238^U dates (FS 3), assuming that the youngest subset of grains are a meaningful age for the depositional age of the ash bed and that older grains record prolonged residence of zircon in the magmatic systems as well as intramagmatic recycling. In the text, all quoted ages of ash beds are weighted mean ^206^Pb/^238^U ages corrected for initial ^230^Th disequilibrium. General chemical abrasion procedures followed the ones described in^79^ modified after^80^. Grains were handpicked and selected for annealing at 900 °C for 48 hours. Grains from each individual sample were chemically abraded for 12 hours in 3 ml Teflon beakers at 210 °C with 12 N HF inside a pressure dissolution vessel. Following chemical abrasion, the grains were then placed inside 3 ml Teflon beakers rinsed and cleaned. Grains were cleaned 6.2 N HCl and 7 N HNO_3_ inside 3 ml Savillex beaker at 80 °C on a hot plate. Total dissolution was done in microcapsules in 12 N HF for 48 hours at 210 °C and spiked using the EARTHTIME ^202^Pb-^205^Pb-^235^U-^233^U and EARTHTIME ^205^Pb-^235^U-^233^U tracer solutions. Conversion to chloride was done by using 6.2 N HCl for 12 hours at 180 °C in the pressure dissolution vessels. 3.1 N HCl was added to the microcapsules before eluting Pb and U using micro columns. The micro columns were first cleaned using four steps alternating 6.2 N HCl and ultrapure H_2_O. Samples were collected in 7 ml Savillex beakers. Data acquisition was done at the Department of Earth Sciences, University of Geneva, Switzerland, using a Thermo Scientific Triton thermal ionisation; mass spectrometer. Each measured ratio was corrected for fractionation using a ^202^Pb/^205^Pb of 0.99989 when the ^202^Pb-^205^Pb-^235^U-^233^U and EARTHTIME was used. When EARTHTIME ^205^Pb-^235^U-^233^U was used Pb fractionation was assumed to be 0.13 ± 0.5% a.m.u (2σ). All common Pb measured was assumed to be from laboratory blank. Uranium was measured as UO_2_^+^ in static mode on Faraday cups equipped with 10^12^ Ω resistors. Auto focusing and peak centring was performed at the beginning of every block, with each block consisting of 20 cycles each. Baselines were monitored on ± 0.5 mass units. ^238^U/^235^U of the sample and blank was assumed to be 137.818 ± 0.045 (2σ)^81^. The oxide correction in U measurements was assumed ^16^OU/^18^OU = 0.002^41^. Uranium decay constant values were used from^82,83^. Raw U-Pb data was reduced using U-Pb Redux and Tripoli^42^ and the data reported in TS.1. Uncertainty and error propagation algorithm used in Redux software is described in^41^. Uncertainties are reported as X/Y/Z; X includes analytical uncertainty, Y includes additional tracer (ET2535or ET535) calibration uncertainty, and Z includes additional ^238^U decay constant uncertainty.

### Bayesian age-depth modelling

In order to assign absolute numerical ages of palaeontological markers, biostratigraphic zone boundaries, to estimate the duration of carbon isotopic fluctuations, and of sedimentation rates, Bayesian statistical interpolation was used using the Bchron package in R studio^84,85^. An age-depth model has been calculated with a 95% confidence uncertainty envelope for every designated stratigraphic depth (10 cm) within the sedimentary column. The model relies on some fundamental assumptions: (1) the subset of U-Pb dates from selected grains as well as their uncertainties are normally distributed, (2) the principle of stratigraphic superposition is not violated. The model assumes that sediment accumulation rate is a random process which implies piecewise monotonic sediment accumulation paths between two dated horizons. These sediments accumulation paths are considered to have Poisson distribution and the thickness of sediment accumulation a gamma distribution. This approach allows for sedimentations rates to vary, in contrast to linear interpolation that considers sedimentations rates to be constant.

### Spline Fitting

To present a statistically valid interpretation of our geochemical data, we fitted the geochemical data points by using a spline-regression model in R Studio. Since the geochemical data appear to have been affected by secondary processes, the main goal of the spline-fitting model is to distinguish between the true geochemical signals from the noise in the data set generated by secondary processes. The model considers each data point as random and normally distributed variables, rather than absolute values. The regression curve should not be understood as the absolute values of the geochemical parameter over time, but rather a main trend in the geochemical data. The code was written and made available for free by Prof. Albert Y. Kim, from the Smith College, MA, USA. The code can be found in the Supplementary Information as well as Prof. Albert Y. Kim’s personal GitHub webpage: https://gist.github.com/rudeboybert/752f7aa1e42faa2174822dd29bfaf959 (last access: 12.01.2019)

### Carbon isotope analysis

The whole-rock powdered samples were decarbonated using 10% HCl acids, weighed 300–500 microgram per sample, and placed in tin microcapsules. The carbon isotope composition of organic matter was determined by flash combustion on a Carlo Erba 1108 elemental analyser connected to a Thermo Fisher Scientific Delta V Plus (Bremen, Germany) isotope ratio mass spectrometer that was operated in the continuous helium flow mode via a Conflo III split interface. The stable carbon isotope compositions were reported in the delta (δ) notation as the per mil (‰) deviations of the isotope ratio relative to Vienna Pee Dee Belemnite limestone (VPDB) standard (δ^13^C_org_ value in ‰ vs. VPDB). The repeatability and intermediate precision of the δ^13^C_org_ analyses, determined by the standard deviation of replicated analyses of laboratory organic standards and unknown samples, were better than 0.1‰ (1 SD). The accuracy of the analyses was checked periodically by analyses of the international reference materials (USGS-24 graphite, IAEA-PEF1 polyethylene foil, and NBS-22 oil).

### Rock Eval pyrolysis

Characterisation; of the organic matter was measured using whole-rock powders on a Rock Eval 6 pyrolysis at the University of Lausanne. Analysed samples weighed 100 to 150 mg. Pyrolysis consists of stepwise heating from 25 °C to 500 °C at a rate of 25 °C/minute. During heating a release of CO and CO_2_ is monitored and graphed by S1, S2, and S3 peaks. S1 peaks are acquired from 25 °C to 300 °C, when hydrocarbon as generated. S2 peaks are monitored from 300 °C to 500 °C representing the sum of all hydrocarbons, and the hydrocarbon potential, and related the amount of hydrogen within the organic matter. The temperature recorded at the peak of S2 is the T_max_. S3 peaks were monitored from 300–390 °C and monitor the amount of CO and CO_2_ being released and relate to the amount of oxygen within the Organic matter. Total Organic content (TOC) is calculated in percent per weight, based the total amount of all carbon components released as pyrolized carbon and residual carbon. Hydrogen Index (HI) (HI, mg HC /g TOC, where HC is hydrocarbons), Oxygen Index (OI, mg CO_2_/g TOC). An in-depth overview of the methodology can be seen in^86^.

### Mercury analysis

Mercury analyses were done by atomic absorption spectrometry designed for Hg analysis on the Zeeman R-915F at the University of Lausanne. Analyses were made by thermal evaporation of whole-rock powdered samples not previously treated. To ensure accuracy, precision, and reproducibility of each measurement the same sample was run at least twice and an average mean value was calculated per sample.

### Re-Os isotopic analysis

Approximately 0.5 g of finely powdered sample was mixed with a ^185^Re-^190^Os tracer solution in a borosilicate Carius tube. After addition of 4 ml 2 M H_2_SO_4_, the Carius tube was frozen in a dry ice/2-propanol mixture. Prior to sealing, 4 ml of 2 M H_2_SO_4_ containing 0.2 g/g dissolved CrO_3_ was added. Samples were digested at 200 °C overnight. Osmium: Carius Tubes tubes were refrozen in a dry ice/2-propanol mixture for opening. Osmium was extracted following a procedure modified after^87^, using a liquid/liquid extraction with CHCl_3_, followed by back extraction into 9 M HBr. The samples were taken to dryness, followed by micro-distillation of the Os^88^. For measurement by N-TIMS, samples were loaded onto 99.999% Pt wire (Materion) with a saturated Ba(OH)_2_ in 0.1 M NaOH solution as activator. Samples were measured at the National Centre for Isotope Geochemistry (NCIG) at University College Dublin on a ThermoScientific Triton as OsO_3_^–^^89,90^. Measurements were performed on the SEM in peak-hopping mode. Interferences of ^187^ReO_3_ on ^187^OsO_3_ were monitored with ^185^ReO_3_ and corrected for. Oxygen corrections were performed using ^17^O/^16^O of 0.0003709 and ^18^O/^16^O of 0.0020449^91^. Mass fractionation was corrected using ^192^Os/^188^Os of 3.083^92^. Long-term reproducibility of measurements was monitored by repeated measurements of the Durham Romil Os Standard solution (DROsS), which yielded a ^187^Os/^188^Os value of 0.16091 ± 0.00016 (n = 56), in good agreement with results from other laboratories (0.160924 ± 0.000004^93^, Durham University; 0.16078 ± 0.00024^94^, University of Alberta). A blank processed alongside the samples for this study yielded 109 fg of Os with a ^187^Os/^188^Os of 0.26. Rhenium: The remainder of the sample was taken to dryness, and redissolved in 15 ml of 5 M NaOH solution. Rhenium was extracted in 15 ml of acetone^95^. After drydown of the acetone, Re was purified over anion resin columns (AG1-X8, 200-400 mesh). Measurements were performed on the Thermo Scientific Neptune at NCIG, using a SIS glass spray chamber. Solutions were doped with W, and mass bias was corrected using a ^184^W/^186^W of 1.0777. Rhenium standard solutions were run at the beginning and end of the analytical session, and additional mass bias corrections were carried out for a ^185^Re/^187^Re value of 0.5974. All corrections were minor. The total Re blank processed alongside the samples was 65 pg.

## Supplementary information

Supplementary Information

## Data Availability

All the raw data can be found in the Supplementary Information.
