# Author Correction: The driving mechanisms of the carbon cycle perturbations in the late Pliensbachian (Early Jurassic)

**DOI:** 10.1038/s41598-021-85194-6

**Published:** 2021-03-10

**Authors:** Luis F. De Lena, David Taylor, Jean Guex, Annachiara Bartolini, Thierry Adatte, David van Acken, Jorge E. Spangenberg, Elias Samankassou, Torsten Vennemann, Urs Schaltegger

**Affiliations:** 1 Department of Earth Sciences, University of Geneva, Geneva, Switzerland; 2 Department of Earth Sciences, University of Oregon, Portland, OR USA; 3 Department of Earth Sciences, University of Oregon, Eugene, OR USA; 4 Faculty of Geosciences and Environmental Sciences, University of Lausanne, Lausanne, Switzerland; 5 Center for Research on Palaeontology – Paris, UMR7207, MNHN, CNRS, SU, Paris, France; 6 Institute of Earth Sciences, University of Lausanne, Géopolis, Lausanne, Switzerland; 7 Irish Centre for Research in Applied Geosciences (iCRAG), UCD School of Earth Sciences, University College Dublin, Dublin, Ireland

Correction to: *Scientific Reports* https://doi.org/10.1038/s41598-019-54593-1, published online 05 December 2019

In the original version of this Article, information on the loading data file format for Bchron was missing from the Supplementary Information. This has now been corrected in the PDF and HTML versions of the paper.

