# Supplementary material for: The driving mechanisms of the carbon cycle perturbations in the late Pliensbachian (Early Jurassic)
Source: Sci Rep. 2019 Dec 5;9:18430. doi: 10.1038/s41598-019-54593-1 (PMC6895128; doi:10.1038/s41598-019-54593-1)
Supplement: Supplementary file 1 — Supplementary Information [file 41598_2019_54593_MOESM1_ESM.pdf]

Supplementary Information

The driving mechanisms of the carbon cycle perturbations in the late Pliensbachian (Early Jurassic)

Luis F. De Lena*, David Taylor, Jean Guex, Annachiara Bartolini, Thierry Adatte, David Van Acken, Jorge Spangenberg, Elias Samankassou, Torsten Vennemann, Urs Schaltegger

1. The tectonic and stratigraphic framework

We have investigated the Nicely and the Suplee formations in the Izee Basin in John Day area in western Oregon, USA. These formations are a part of a marine sedimentary megasequence within the early Mesozoic Izee terrane in the Blue Mountains Province[1]. Besides the Izee terrane the Province includes the Olds Ferry terrane, Baker, and Wallowa terranes and represents an important record of crustal Mesozoic evolution of the North American Cordillera from the Paleozoic to Cretaceous[2,3].

The Nicely Fm. is composed mainly of organic-rich black mudstones with intercalated sandstones and carbonates (FS.6). The lack of thick-shelled, shallow-water benthic faunas along with mostly thinly- and parallel-laminated mudstone in the Nicely Fm. suggests it was deposited mostly in a comparatively deeper-water offshore setting. The Suplee Fm., with its diverse thick-shelled benthic fauna, is composed mainly of calcareous siltstones, conglomerates and sandstones reflecting a shallow, more proximal marine environment. The formations span the Late Pliensbachian and contain the Kunae and the Carlottense ammonite zones, which are equivalent to the Margaritatus and to Spinatum ammonite zones in western Europe.

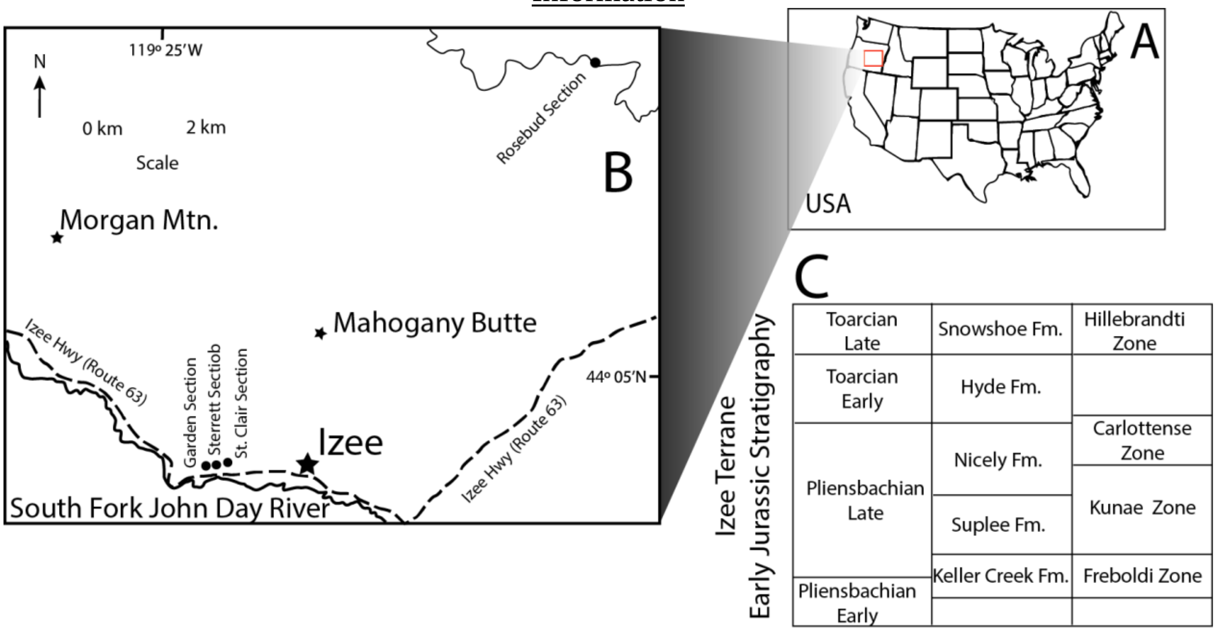

FS. 1 – Location map of the studied sections. A) Location of the state of Oregon, USA; B) the geographical location of the sampled sections; C) Simplified stratigraphy of the Early Jurassic of the Izee Terrane.

2. Cross-continental correlation between North American and Northwestern European late Pliensbachian ammonite zones

The presence of *Fanninoceras* low in the Suplee Formation indicates that the formation is referable to the Kunae Zone [4,5](FS. 2). *Prodactylioceras italicum* (FS. 2) occurs near the top of the Suplee Fm. As the species occurs no higher than the Stokesi Subzone in Europe [6–9], we propose that the Suplee Fm. is largely correlative with that Subzone. A caveat is that the first *Fanninoceras* (Fig. 1, FS. 2) (hence Kunae Zone) may correlate with the uppermost Davoei Zone [4,5,10]. There is an example of *Fuciniceras* (*Paltarpites*) from the topmost Suplee Formation in the vicinity of the Rosebud section, in beds we herein correlate with the Stokesi Subzone (Fig. 1). *Fuciniceras* (*Paltarpites*) in Europe is not known below the Gibbosus Subzone. Considering that *F.* (*Paltarpites*) is merely an involute, closely ribbed *Fuciniceras*, we suggest that the "involute stage-of-evolution" was reached earlier in North American than in Europe. The correlatives of the Subnodosus and Gibbosus subzones, therefore, occur in the lower part of the Nicely Formation, where they are bracketed by ammonites equivalent to the Northwest European Stokesi Subzone (below) and the North American Carlottense Zone (FS.1, FS. 2).

The Kunae zone, thus, encompasses the Suplee and the lower part of the Nicely Formations. Some of the species of *Fanninoceras* have range overlaps between the zones. *Fanninoceras carlottense* (FS.2) occurs typically in the Carlottense Zone, but[11] report that it rarely occurs in the subjacent Kunae Zone. Conversely, while *Fanninoceras fannini* dominates in the Kunae Zone, it uncommonly occurs higher [5,11,12]. *Fontanelliceras,* which ranges down into the Spinatum Zone in Europe persists into the Kunae Zone in North America [4,11,13]. On the other hand, *Lioceratoides* and *Tiltoniceras* have not been reported below the Carlottense Zone. The Carlottense Zone is taken to begin with the appearance of *Lioceratoides*, which in the Garden of Concretions section (FS. 2) is just above the base of the upper Nicely. *Fanninoceras fannini* and *F.* cf. *fontanellense* therefore range lower while *Fanninoceras fannini* occurs (FS.2) in the Carlottense Zone. The fauna from uppermost one meter of the Nicely Fm. at the Sterrett, Section is correlated northwest European Hawskerense Subzone of latest Pliensbachian age (Fig. 1, FS. 2). The Oregon examples of *Emaciaticeras* are closest to *E. lottii* and differ only in that the projected ribbing on the venter is more pronounced. The species suggests a correlation with the Hawskerense Subzone of the Spinatum Zone (= Elisa Subzone of Emaciatum Zone) since in Europe and North Africa the species is reported from that level [14–16]. The presence of *Amaltheus* (*Psuedoamaltheus*) sp. further confirms a Pliensbachian age for the fauna since no representatives of the genus have been found in the Toarcian.

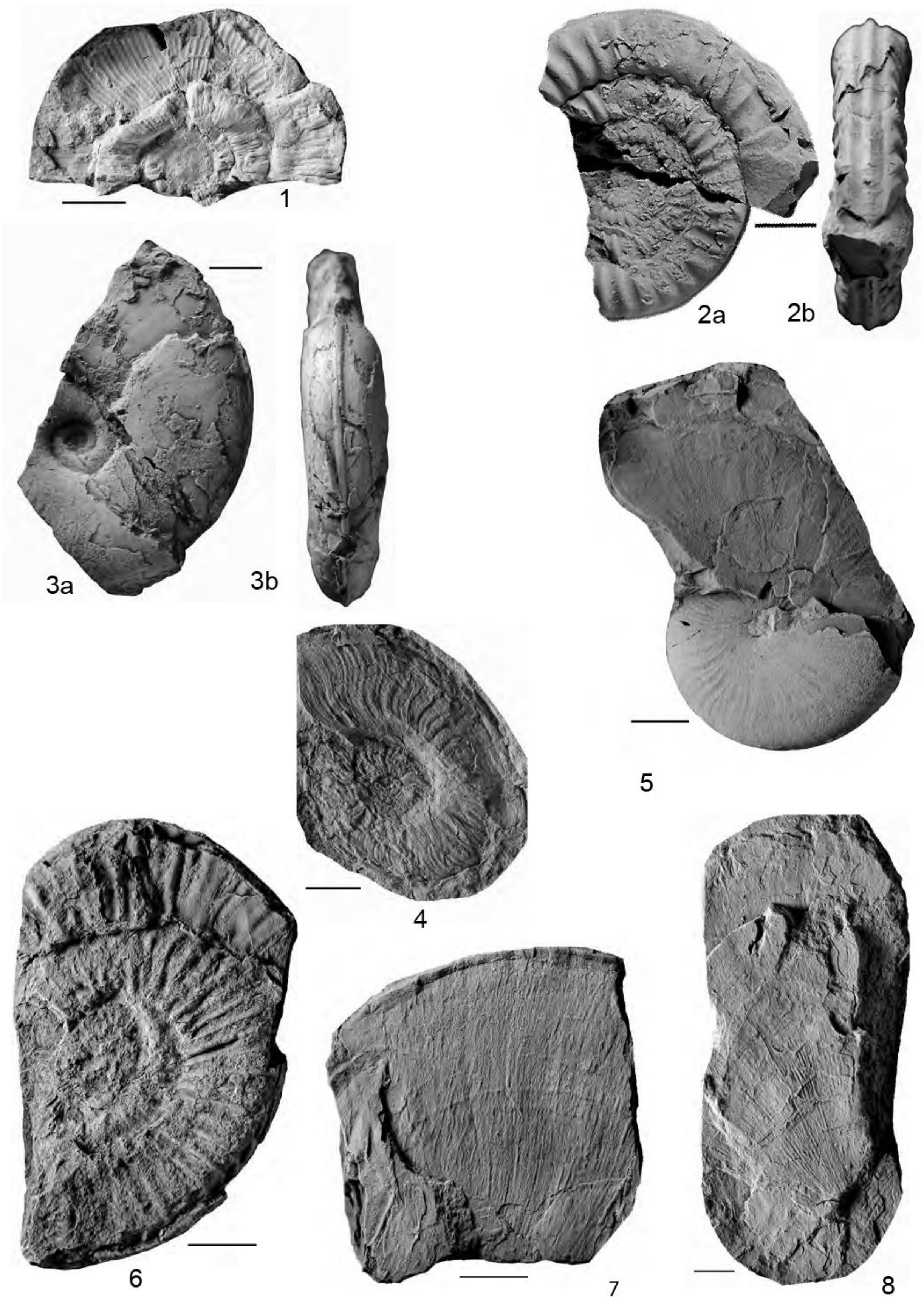
1
2a
2b
3a
3b
4
5
6
7
8

FS.2 – Ammonites: 1.) *Prodactylioceras italicum* ; 2 a, b.) *Fontanelliceras cf. fontanellense*; 3 a, b.) *Tiltoniceras antiquum*; 4.) *Lioceratoides aff. silvestrii*; 5. *Fanninoceras carlottens*e; 6.) *Emacia ticeras aff. lotti*; 7.) *Amaltheus (Pseudoamaltheus)* sp.; 8.) *Amaltheus (Pseudoamaltheus)* sp. Scale bar = 1 cm.

3. U-Pb geochronology

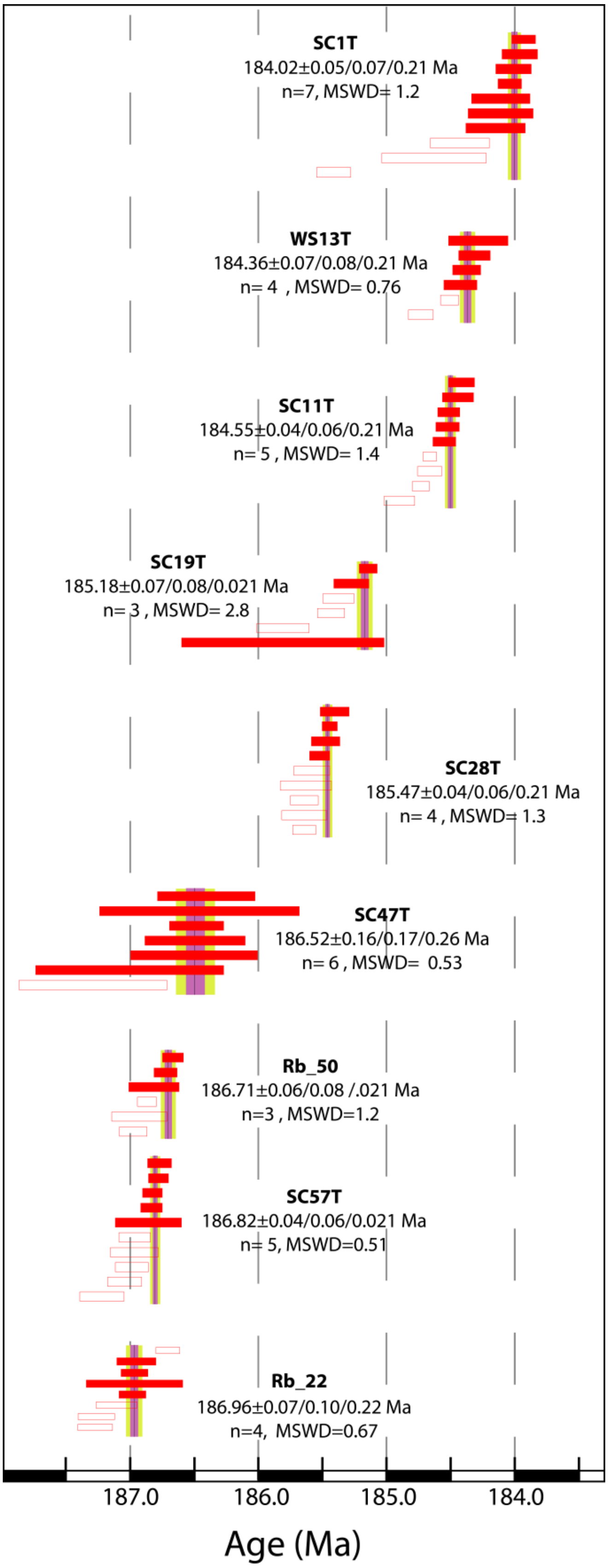

FS. 3 – All U-Pb single grain analyses are in either colored or uncolored red bars. Colored bars are dates used in the weighted mean, and uncolored bars and older grains which were left out of the weighted mean, and were interpreted to record prolonged residence of zircon in the magmatic systems as well as intramagmatic recycling. Purple bars represent the 1σ, and yellow bars represent 2σ precision, respectively. All ages are reported as X/Y/Z, where X includes analytical uncertainty only, Y includes analytical and tracer calibration uncertainty, Z includes analytical, tracer calibration and ^{238}U decay constant uncertainty.

4. Supplementary Rock Eval Data

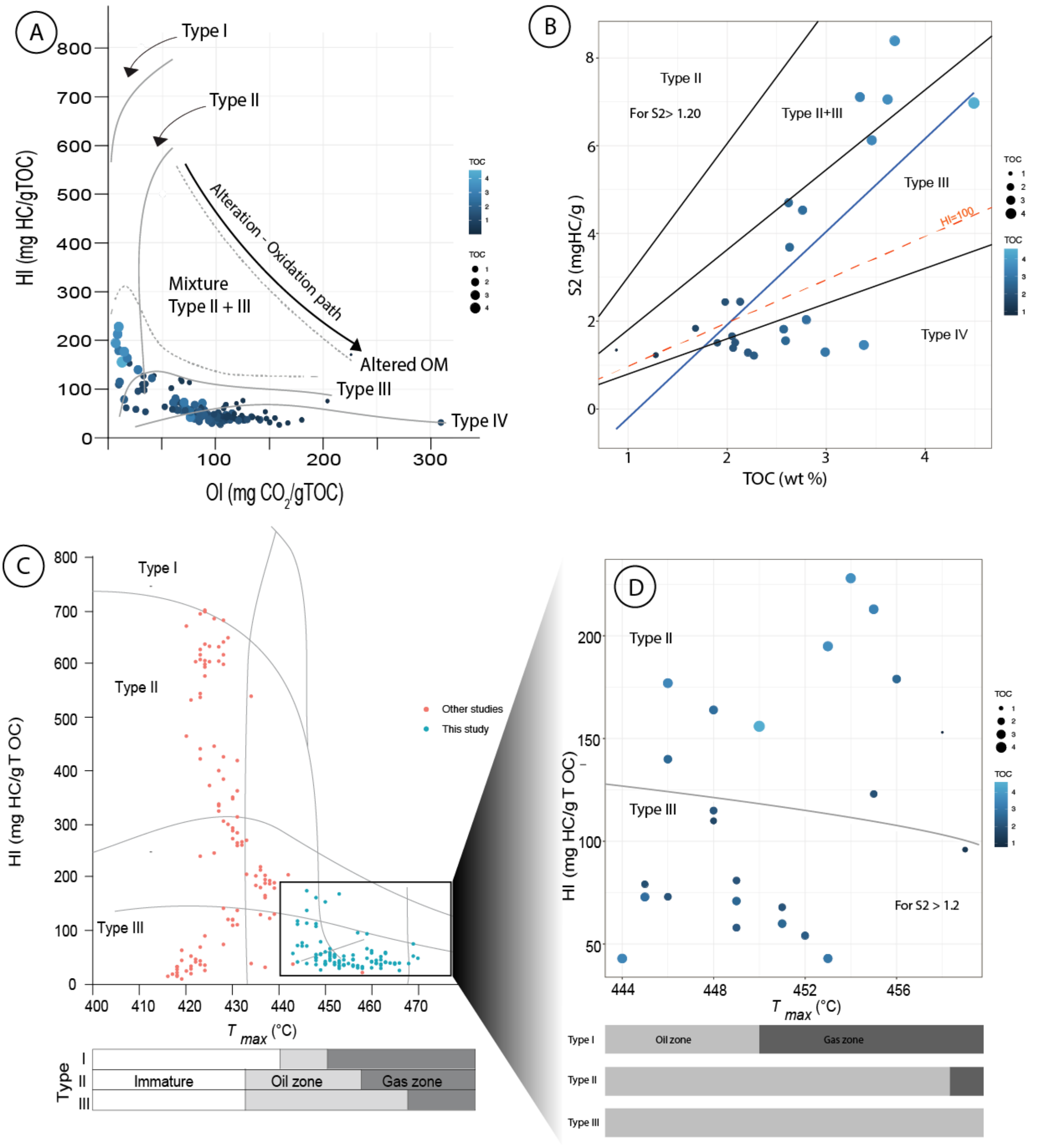

FS.4 – A) Van Krevelen diagram (HI vs OI), which suggests that the majority of the OM in the Nicely Fm is of type III to type IV, with a mixture of type II and type III OM. There appears to be a positive correlation between the oxidation path of OM in the Van Krevelen diagram and TOC values, which is an indication that secondary processes have affected the OM in the Nicely Fm. B) S2 vs TOC plot for data points with S2 >1.2, notably those in the period II of the late Pliensbachian event where there is a shift in the S2 peak (See Fig. 2). C) HI vs T_{max}. Red data

points are from [17–21] green data points are from this study. The HI vs T_{max} plot shows that the OM from the Nicely and Suplee formations is mature to over-mature, whereas data from in North-western Europe show that the OM is immature to mature. D) HI vs T_{max} for data points with S2 >1.2. It is obvious that values over 460°C are not confident readings of T_{max} values as they do not appear in the plot, which is the result of such low TOC values (<0.5%). Additionally, FS.4D shows that a small part of the OM is in fact type II and type III and show no trend with varying TOC values. In summary, FS. 4 shows that the majority of the OM in the Nicely Fm. is mature to over-mature, with HI and OI values possibly overprinted by diageneses, burial and secondary processes; however, mainly type III OM. This is also observed from FS. 4A where the oxidation-alteration path shows a correlation with the amount of TOC in the sample.

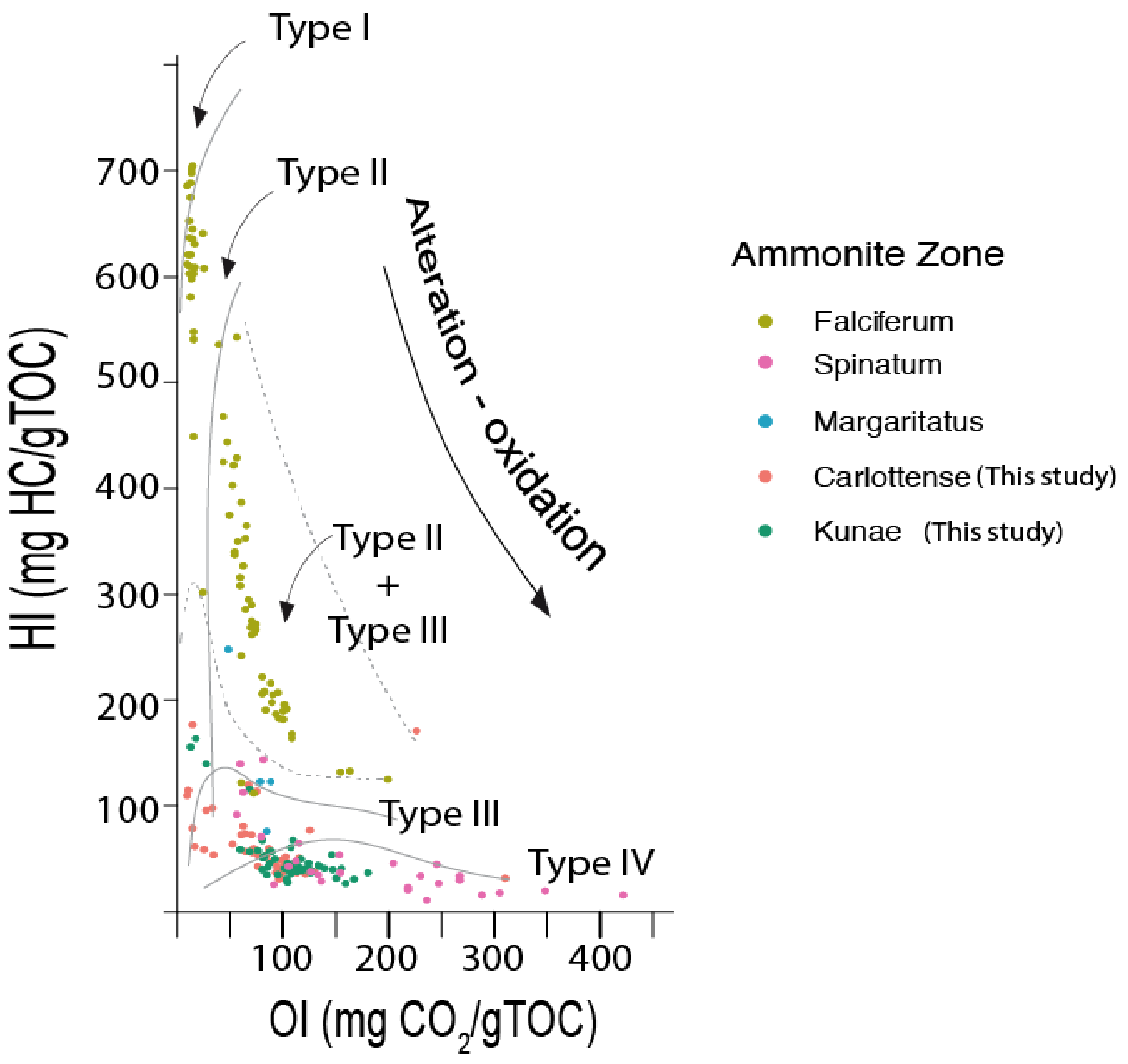

FS. 5 – HI and OI data for the Kunae and Carlottense Zones from this study; data for the Falciferum, Spinatum, and Margaritatus Zones are taken from[17–21]. Note that the characterization

of OM as mainly type III from the Nicely Fm. (Kunae and Carlottense Zones) is in accordance with their counterparts (Margaritatus and Spinatum Zones) from studies in western Tethys, and differ considerably with respect to the OM deposited during the early Toarcian OAE.

5. Age-depth modelling - Bchron code

```
mydata2 = read.table(file='FILE DESTINATION.txt', header=TRUE)
GlenOut = Bchronology(ages=mydata2$ages,
                ageSds=mydata2$ageSds,
                calCurves=mydata2$calCurves,
                positions=mydata2$position,
                positionThicknesses=mydata2$thickness,
                ids=mydata2$id,
                predictPositions=seq(0,8400,by=10),iterations = 10000)
plot(GlenOut,main="PliensBchron",xlab='Age (Ma)',ylab='Depth (cm)',las=1)
summary(GlenOut)
summary(GlenOut, type='convergence')
summary(GlenOut, type='outliers')


Output <- cbind(apply(GlenOut$thetaPredict, 2, quantile,  probs = c(.0025)),
    apply(GlenOut$thetaPredict, 2, quantile,  probs = c(.5)),
    apply(GlenOut$thetaPredict, 2, quantile,  probs = c(.975)))
write.csv(Output, file = 'whatever.csv', quote=FALSE, row.names = FALSE)


acc_rate = summary(GlenOut, type = 'acc_rate')
plot(acc_rate[,'age_grid'], acc_rate[,'50%'], type='l', ylab = 'cm per year', xlab = 'Age (k cal
years BP)', ylim = range(acc_rate[,-1]))
lines(acc_rate[,'age_grid'], acc_rate[,'2.5%'], lty='dotted')
lines(acc_rate[,'age_grid'], acc_rate[,'97.5%'], lty='dotted')
sed_rate = summary(GlenOut, type = 'sed_rate', useExisting = FALSE)
plot(sed_rate[,'position_grid'], sed_rate[,'50%'], type='l', ylab = 'Years per cm', xlab = 'Depth
(cm)', ylim = range(sed_rate[,-1]))
lines(sed_rate[,'position_grid'], sed_rate[,'2.5%'], lty='dotted')
lines(sed_rate[,'position_grid'], sed_rate[,'97.5%'], lty='dotted')

write.csv(sed_rate, file = ' PliensBchron _sed_rates.csv', quote=FALSE, row.names =
FALSE)
write.csv(GlenOut, file = ' PliensBchron _sed_rates.csv', quote=FALSE, row.names = FALSE
```

6. Spline fitting code

```
DATATABLE<-read.csv(file.choose(), header=TRUE)
attach(OSRE)

# Fit spline
smooth.spline(DATATABLE$StratDepth, DATATABLE $VARIABLE, df=20) %>%
  broom::augment() %>%
  ggplot(aes(x=StratDepth)) +
  theme_bw() +
  geom_point(aes(y=VARIABLE)) +
  geom_line(aes(y=.fitted, col="blue"))
```

https://gist.github.com/rudeboybert/752f7aa1e42faa2174822dd29bfaf959
last access: 12.01.2019

7. Field Figures

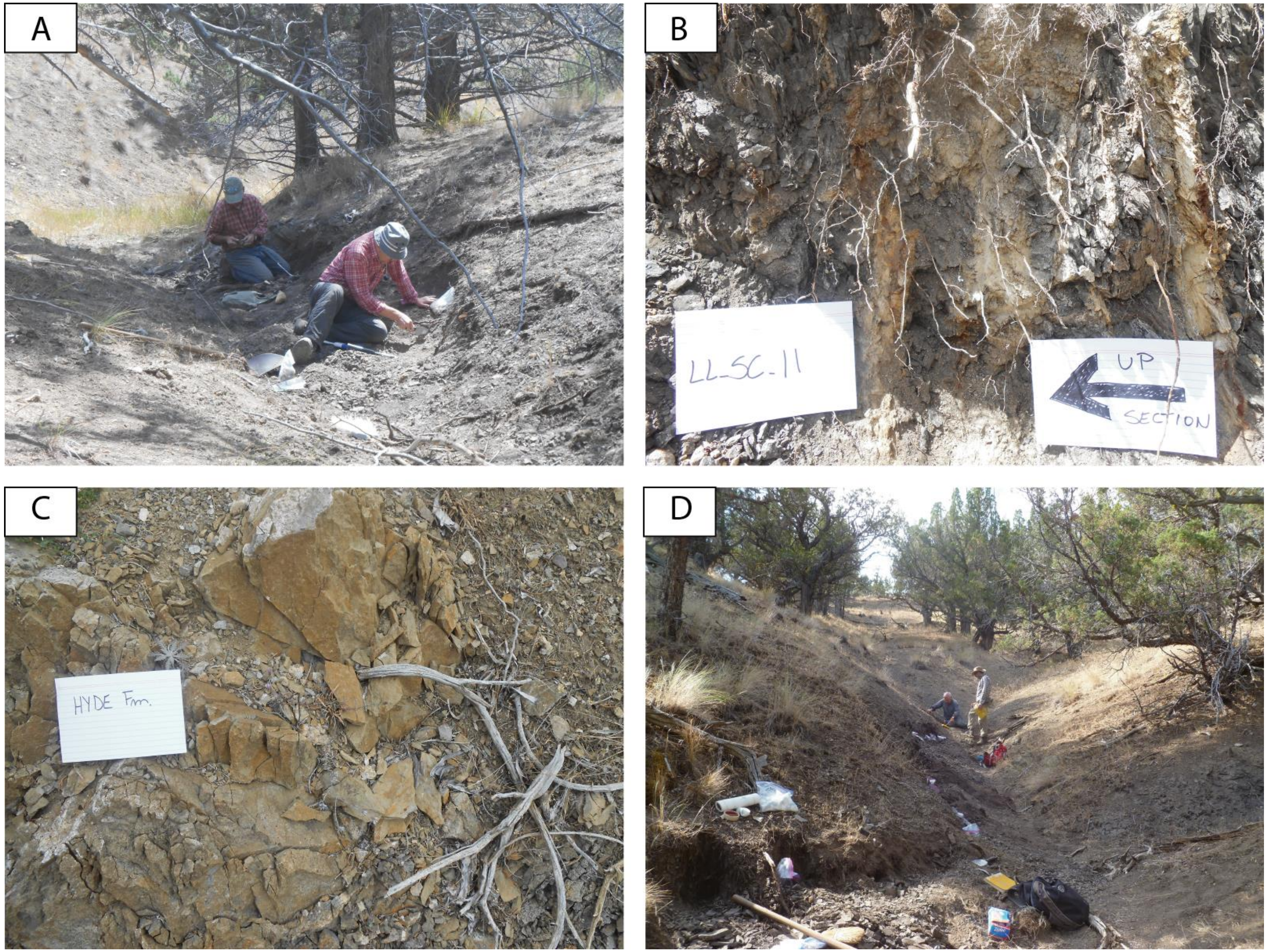

FS. 6 – Field Figures. A) Photo of the St. Clair section with a view of the lower Nicely Fm. B) Photo of the of the ash beds in bed11 in the St. Clair section. C) Field phot of the Hyde Fm. D) Field photo of the upper St. Clair section, view of the upper Nicely.

8. Stratigraphic logs and ammonite distribution

Rosebud section

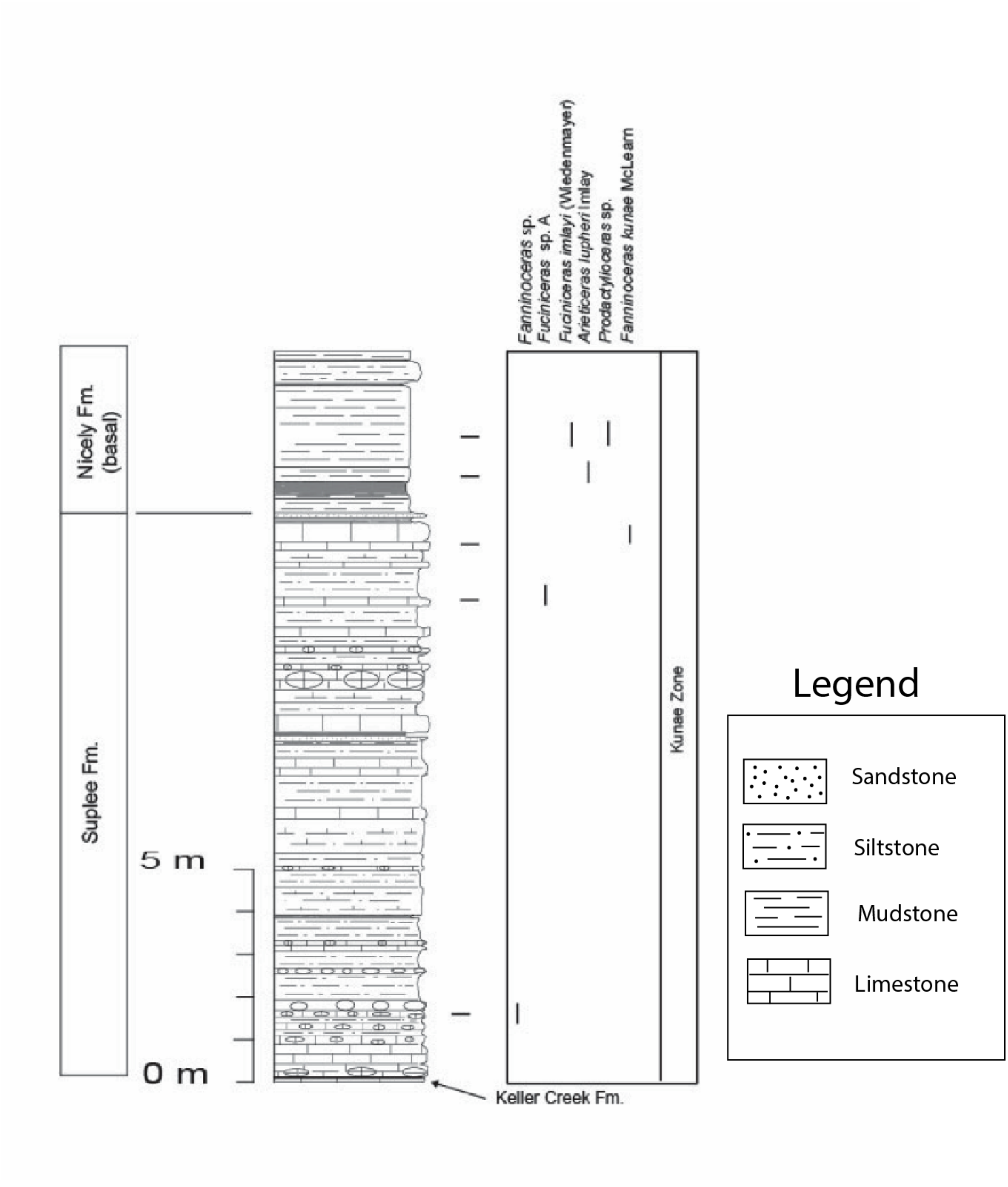

Garden of Concretion section

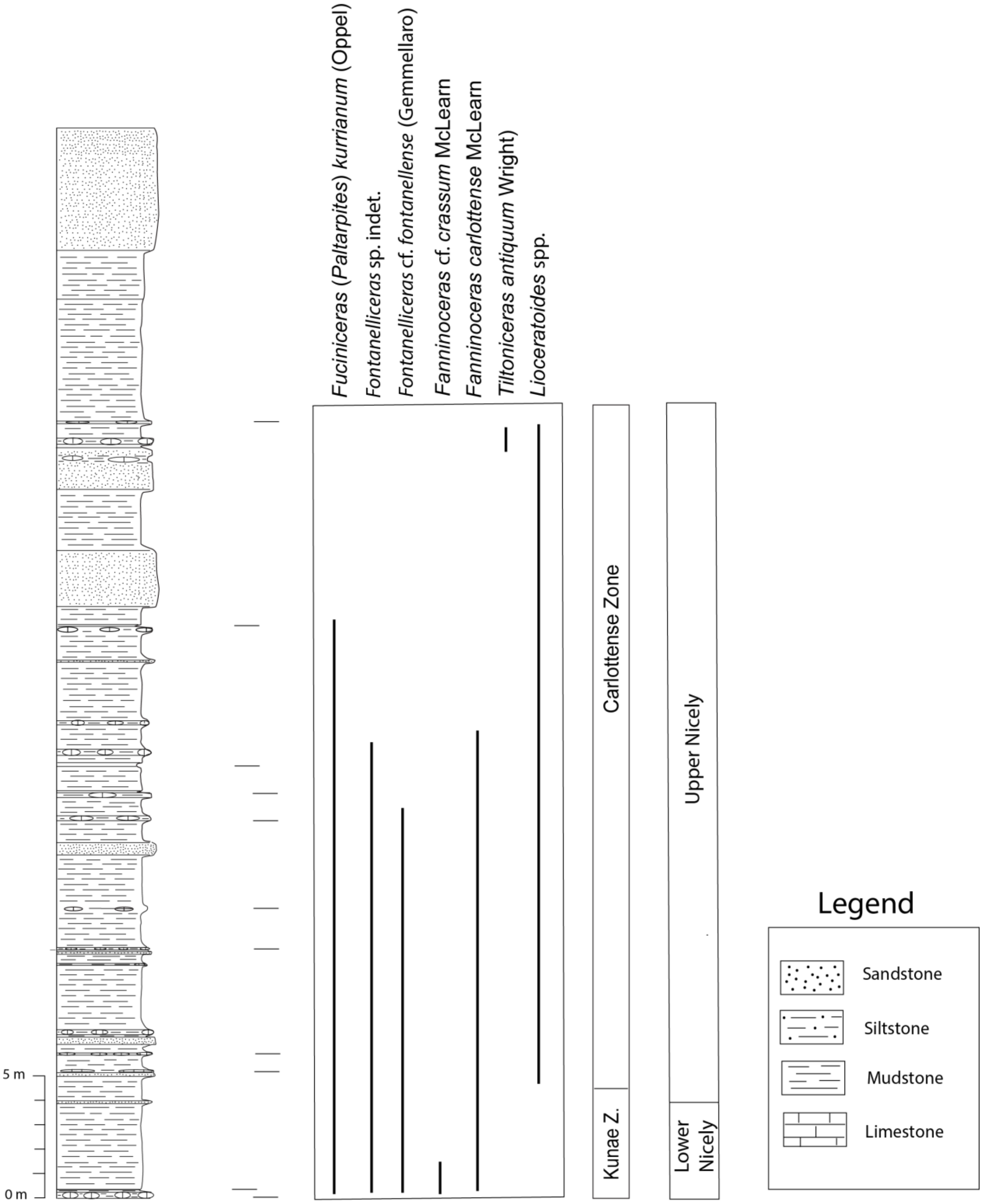

Saint Clair section

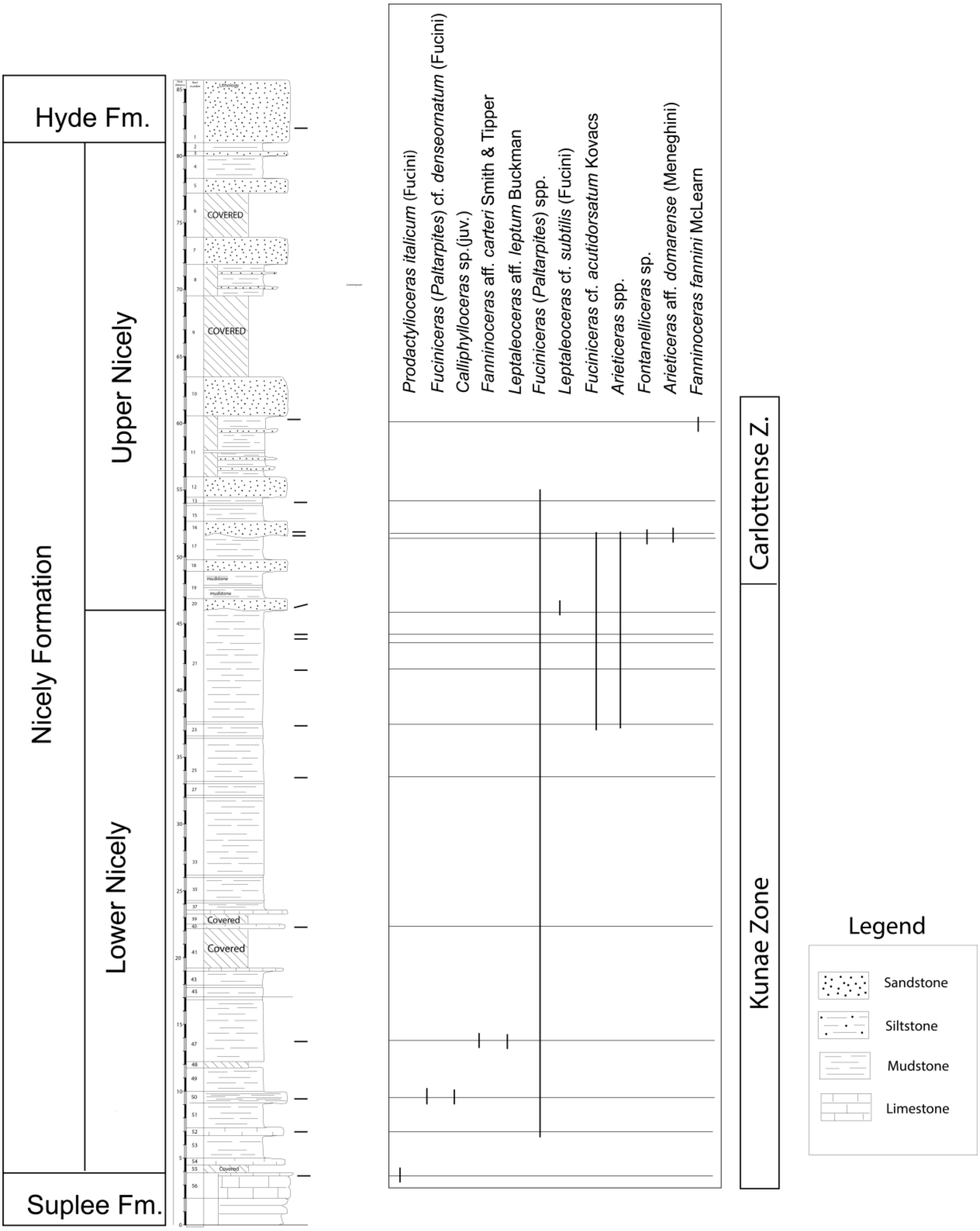

Sterrett Section

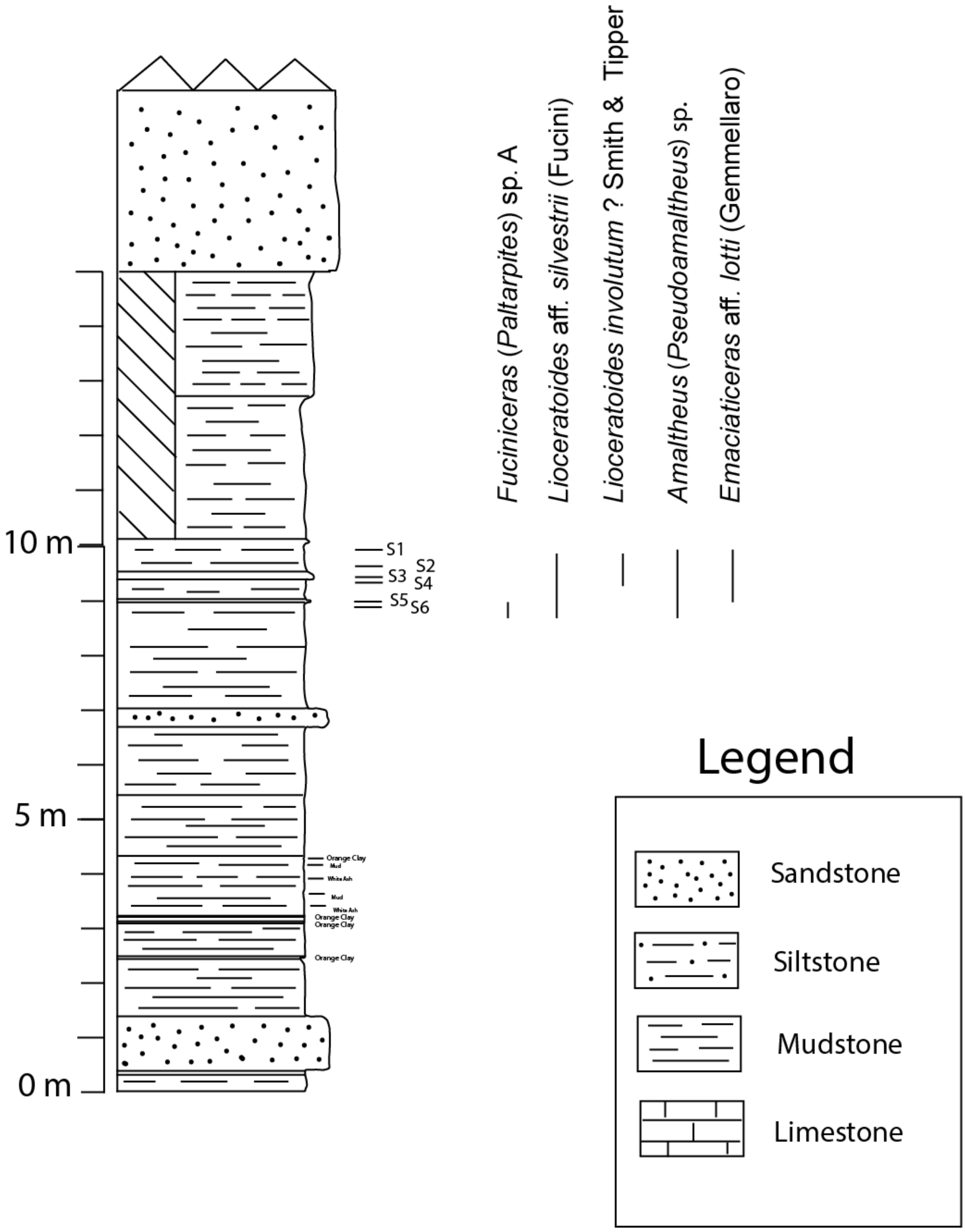

9. Data tables

U-Pb geochronology data table TS.1

	206Pb/ 238U <Th> (Ma) **a**	±2σ abs	207Pb/ 235U (Ma) **b**	±2σ abs	207Pb/ 206Pb (Ma) **b**	±2σ abs	Corr. coef.	Th/ U **c**	Pb* (pg) **d**	Pbc (pg) **e**	206Pb/ 238U (ratios) **f**	±2σ %	207Pb/ 235U (ratios) **f**	±2σ %	207Pb/ 206Pb (ratios) **f**	±2σ %
**SC1T**																
**z1**	**184.131**	**0.242**	**184.162**	**2.206**	**185.664**	**28.169**	**0.77**	**0.65**	**5.67**	**0.64**	**0.028961**	**0.133219**	**0.198861**	**1.309565**	**0.049822**	**1.209403**
z2	184.471	0.242	185.941	2.318	205.731	29.466	0.73	0.73	5.23	0.62	0.029016	0.132882	0.200964	1.364525	0.050254	1.269754
**z3**	**184.026**	**0.142**	**183.926**	**0.517**	**183.851**	**5.878**	**0.76**	**0.41**	**27.85**	**0.52**	**0.028943**	**0.078483**	**0.198583**	**0.307055**	**0.049783**	**0.250298**
z4	185.522	0.141	185.314	0.901	183.815	11.236	0.68	0.52	9.25	0.41	0.029183	0.077013	0.200222	0.531846	0.049783	0.481329
**z5**	**184.132**	**0.268**	**184.233**	**2.471**	**186.687**	**31.500**	**0.79**	**0.53**	**3.63**	**0.49**	**0.028961**	**0.147484**	**0.198945**	**1.466430**	**0.049844**	**1.352749**
**z7**	**183.939**	**0.095**	**182.350**	**0.881**	**163.012**	**11.647**	**0.60**	**0.43**	**11.26**	**0.51**	**0.028930**	**0.052542**	**0.196724**	**0.527816**	**0.049341**	**0.497115**
z8	184.688	0.435	186.698	4.431	213.227	56.344	0.72	0.84	2.06	0.45	0.029051	0.238878	0.201859	2.598225	0.050417	2.431925
**z9**	**184.176**	**0.244**	**182.843**	**2.463**	**166.829**	**32.229**	**0.71**	**0.47**	**6.43**	**0.92**	**0.028968**	**0.134444**	**0.197305**	**1.472271**	**0.049421**	**1.379022**
**z10**	**183.974**	**0.146**	**182.217**	**1.501**	**160.717**	**19.882**	**0.64**	**0.35**	**2.79**	**0.22**	**0.028935**	**0.080589**	**0.196566**	**0.899742**	**0.049292**	**0.849384**
**z13**	**184.057**	**0.094**	**182.057**	**0.628**	**157.304**	**7.933**	**0.77**	**0.64**	**6.86**	**0.17**	**0.028949**	**0.051307**	**0.196379**	**0.376881**	**0.049220**	**0.337406**
**WS13T**																
**z1**	**184.422**	**0.134**	**183.148**	**0.677**	**167.878**	**8.452**	**0.63**	**0.61**	**9.60**	**0.19**	**0.029008**	**0.073757**	**0.197665**	**0.403796**	**0.049444**	**0.360377**
**z2**	**184.302**	**0.129**	**183.430**	**0.658**	**173.431**	**8.422**	**0.51**	**0.38**	**11.84**	**0.32**	**0.028987**	**0.071044**	**0.197998**	**0.391999**	**0.049562**	**0.359438**
z4	184.510	0.075	184.024	0.335	178.941	4.157	0.58	0.54	17.49	0.25	0.029021	0.041081	0.198699	0.199083	0.049679	0.175360
z7	184.752	0.101	183.800	0.501	172.697	6.021	0.77	0.65	10.17	0.19	0.029060	0.055211	0.198434	0.297885	0.049546	0.255962
**z9**	**184.382**	**0.102**	**185.273**	**0.950**	**197.840**	**12.342**	**0.56**	**0.43**	**11.18**	**0.58**	**0.029000**	**0.056248**	**0.200174**	**0.560978**	**0.050084**	**0.530258**
**z10**	**184.276**	**0.246**	**182.600**	**2.639**	**162.133**	**34.818**	**0.69**	**0.53**	**2.67**	**0.39**	**0.028984**	**0.135272**	**0.197018**	**1.579362**	**0.049322**	**1.488600**
**SC11T**																
**z2**	**184.460**	**0.106**	**184.515**	**0.377**	**186.437**	**4.271**	**0.75**	**0.36**	**33.74**	**0.33**	**0.029013**	**0.058448**	**0.199278**	**0.223207**	**0.049839**	**0.180597**
**z3**	**184.576**	**0.094**	**184.622**	**0.420**	**186.436**	**5.260**	**0.53**	**0.35**	**27.13**	**0.48**	**0.029031**	**0.051743**	**0.199405**	**0.248809**	**0.049839**	**0.223611**
z4	184.797	0.071	184.723	0.560	184.919	7.264	0.55	0.57	33.00	0.98	0.029067	0.038359	0.199524	0.331385	0.049806	0.310257
z5	184.725	0.052	184.820	0.187	187.240	2.508	0.23	0.39	36.57	0.19	0.029055	0.028231	0.199638	0.110690	0.049856	0.102769
z7	184.725	0.100	184.983	0.422	189.476	5.056	0.66	0.38	23.97	0.52	0.029055	0.054815	0.199830	0.249728	0.049904	0.214861
**z8**	**184.604**	**0.091**	**184.328**	**0.351**	**181.982**	**3.683**	**1.01**	**0.40**	**24.04**	**0.34**	**0.029036**	**0.049649**	**0.199056**	**0.208440**	**0.049744**	**0.154725**
z21	184.977	0.126	184.312	0.991	177.007	12.884	0.56	0.39	6.92	0.38	0.029095	0.069038	0.199039	0.587796	0.049637	0.551519

**z24**	**184.567**	**0.089**	**183.948**	**0.761**	**177.072**	**9.962**	**0.56**	**0.77**	**16.80**	**0.64**	**0.029032**	**0.048522**	**0.198609**	**0.452576**	**0.049639**	**0.425961**
**z25**	**184.489**	**0.128**	**183.112**	**1.331**	**166.604**	**17.597**	**0.62**	**0.36**	**4.50**	**0.33**	**0.029017**	**0.070231**	**0.197622**	**0.794552**	**0.049417**	**0.752438**
**SC19T**																
**z1**	**185.143**	**0.074**	**185.085**	**0.344**	**185.460**	**3.909**	**0.91**	**0.63**	**18.32**	**0.18**	**0.029123**	**0.040058**	**0.199952**	**0.203587**	**0.049818**	**0.164730**
z4	185.389	0.130	184.954	1.376	180.523	17.890	0.67	0.59	11.94	0.91	0.029162	0.070764	0.199796	0.813594	0.049712	0.766970
**z5**	**185.284**	**0.146**	**184.614**	**1.116**	**177.162**	**13.861**	**0.86**	**0.62**	**6.27**	**0.29**	**0.029145**	**0.079693**	**0.199395**	**0.661384**	**0.049641**	**0.593524**
z7	185.453	0.111	184.490	0.793	173.299	10.441	0.43	0.57	9.27	0.27	0.029172	0.060742	0.199248	0.470232	0.049559	0.446261
z11	185.851	0.218	186.441	2.282	195.041	29.471	0.63	0.57	3.18	0.39	0.029236	0.119018	0.201555	1.339943	0.050023	1.267527
**z13**	**185.854**	**0.844**	**188.543**	**9.539**	**223.446**	**120.315**	**0.76**	**0.62**	**1.96**	**1.05**	**0.029236**	**0.461118**	**0.204044**	**5.543543**	**0.050640**	**5.203031**
**SC24T**																
**z1**	**183.782**	**0.137**	**183.065**	**0.552**	**175.031**	**7.188**	**0.39**	**0.38**	**27.32**	**0.86**	**0.028904**	**0.075680**	**0.197566**	**0.329246**	**0.049596**	**0.306402**
z2	184.522	0.281	183.262	2.831	168.242	36.805	0.75	0.44	7.68	1.26	0.029023	0.154813	0.197799	1.688516	0.049451	1.575340
z3	184.155	0.221	185.526	2.161	204.142	27.606	0.72	0.57	20.68	2.55	0.028965	0.121794	0.200472	1.274370	0.050220	1.189198
**z4**	**183.892**	**0.130**	**183.856**	**0.511**	**184.555**	**6.217**	**0.60**	**0.49**	**28.92**	**0.72**	**0.028923**	**0.071481**	**0.198499**	**0.303910**	**0.049799**	**0.264973**
z5	184.342	0.254	180.839	2.162	136.456	28.157	0.78	0.57	11.28	1.15	0.028995	0.139994	0.194944	1.304968	0.048785	1.197987
**z7**	**183.921**	**0.149**	**183.977**	**0.743**	**185.917**	**9.141**	**0.65**	**0.37**	**13.91**	**0.51**	**0.028927**	**0.082142**	**0.198643**	**0.441449**	**0.049828**	**0.391260**
**z8**	**184.055**	**0.266**	**183.140**	**1.605**	**172.564**	**19.840**	**0.77**	**0.37**	**12.63**	**1.07**	**0.028948**	**0.146873**	**0.197655**	**0.957629**	**0.049543**	**0.849439**
**z9**	**183.900**	**0.138**	**182.612**	**0.591**	**167.199**	**6.614**	**0.94**	**0.35**	**27.05**	**0.50**	**0.028923**	**0.076306**	**0.197032**	**0.353754**	**0.049429**	**0.281242**
**z10**	**183.918**	**0.114**	**184.016**	**0.532**	**186.488**	**6.886**	**0.41**	**0.38**	**19.31**	**0.55**	**0.028926**	**0.062980**	**0.198688**	**0.316092**	**0.049840**	**0.294016**
**SC28T**																
z1	185.665	0.095	184.939	0.906	176.910	11.837	0.58	0.31	6.35	0.33	0.029205	0.051853	0.199779	0.535982	0.049635	0.506535
**z3**	**185.486**	**0.117**	**182.390**	**0.951**	**143.691**	**12.684**	**0.50**	**0.36**	**3.13**	**0.13**	**0.029176**	**0.063723**	**0.196771**	**0.569737**	**0.048936**	**0.539601**
z11	185.665	0.186	185.833	2.008	189.173	25.928	0.69	0.39	4.94	0.58	0.029205	0.101581	0.200836	1.182314	0.049897	1.113816
**z12**	**185.451**	**0.060**	**185.046**	**0.415**	**181.094**	**5.443**	**0.41**	**0.36**	**8.34**	**0.11**	**0.029171**	**0.032461**	**0.199905**	**0.245047**	**0.049724**	**0.231324**
**z13**	**185.536**	**0.083**	**185.659**	**0.578**	**188.446**	**7.062**	**0.84**	**0.33**	**9.96**	**0.25**	**0.029184**	**0.045204**	**0.200630**	**0.340391**	**0.049882**	**0.301746**
z14	185.664	0.115	184.918	1.175	176.622	15.315	0.64	0.34	6.10	0.42	0.029205	0.062659	0.199754	0.695221	0.049629	0.655887
z15	185.652	0.211	183.073	2.142	151.139	28.147	0.70	0.37	4.24	0.53	0.029203	0.115148	0.197576	1.278916	0.049091	1.200798
**z16**	**185.411**	**0.118**	**183.690**	**1.013**	**162.840**	**13.087**	**0.69**	**0.36**	**4.90**	**0.19**	**0.029164**	**0.064775**	**0.198304**	**0.602647**	**0.049337**	**0.558798**
z18	185.603	0.149	185.115	1.507	180.110	19.276	0.80	0.37	6.57	0.49	0.029195	0.081261	0.199987	0.890616	0.049703	0.826392
**SC47T**																
**z3**	**187.064**	**0.785**	**185.673**	**8.685**	**169.124**	**112.380**	**0.74**	**0.59**	**3.78**	**1.83**	**0.029429**	**0.426454**	**0.200646**	**5.118181**	**0.049470**	**4.811875**
**z6**	**186.532**	**0.528**	**179.981**	**5.897**	**95.987**	**79.846**	**0.72**	**0.45**	**1.16**	**0.37**	**0.029344**	**0.287396**	**0.193934**	**3.575337**	**0.047955**	**3.372756**
**z7**	**186.509**	**0.224**	**184.848**	**1.979**	**164.850**	**26.344**	**0.40**	**0.44**	**2.37**	**0.25**	**0.029340**	**0.121966**	**0.199671**	**1.170865**	**0.049380**	**1.126663**
**z9**	**186.522**	**0.417**	**183.274**	**4.625**	**142.831**	**60.913**	**0.74**	**0.34**	**1.39**	**0.37**	**0.029342**	**0.227079**	**0.197814**	**2.758213**	**0.048917**	**2.595399**
**z11**	**186.430**	**0.404**	**185.890**	**3.452**	**180.169**	**44.238**	**0.64**	**0.55**	**1.49**	**0.28**	**0.029328**	**0.219922**	**0.200903**	**2.032162**	**0.049705**	**1.897787**
z13	189.463	6.069	212.282	67.013	474.715	721.027	0.75	0.65	0.66	2.49	0.029813	3.254310	0.232526	34.982547	0.056593	32.600603
z14	187.966	1.203	169.100	13.213	-86.196	196.600	0.73	0.47	0.60	0.44	0.029573	0.650143	0.181209	8.482397	0.044461	8.022568

**z15**	**186.487**	**0.828**	**187.379**	**7.907**	**199.784**	**99.384**	**0.78**	**0.49**	**1.98**	**0.86**	**0.029337**	**0.450930**	**0.202665**	**4.621352**	**0.050126**	**4.279341**
**SC57T**															
z5	186.992	0.131	186.703	0.887	184.178	11.190	0.61	0.53	10.32	0.45	0.029418	0.070966	0.201865	0.520133	0.049791	0.479357
z6	187.267	0.178	187.441	1.595	190.757	20.341	0.63	0.56	8.27	0.68	0.029462	0.096605	0.202739	0.932061	0.049931	0.873828
z7	187.079	0.139	186.758	0.918	183.826	11.671	0.55	0.54	14.88	0.72	0.029432	0.075120	0.201930	0.538268	0.049783	0.500058
z8	186.998	0.200	186.752	1.725	184.796	21.686	0.76	0.49	5.09	0.46	0.029418	0.108598	0.201923	1.011244	0.049804	0.930673
**z10**	**186.884**	**0.270**	**186.182**	**2.753**	**178.451**	**35.293**	**0.73**	**0.48**	**5.66**	**0.89**	**0.029400**	**0.146582**	**0.201249**	**1.618152**	**0.049668**	**1.513453**
**z12**	**186.788**	**0.096**	**184.841**	**0.607**	**161.152**	**7.856**	**0.50**	**0.56**	**9.46**	**0.28**	**0.029385**	**0.051896**	**0.199662**	**0.358937**	**0.049302**	**0.334339**
z13	187.022	0.136	186.374	1.382	179.332	17.810	0.67	0.48	8.60	0.67	0.029422	0.073865	0.201476	0.811896	0.049687	0.763364
**z14**	**186.847**	**0.081**	**187.114**	**0.649**	**191.625**	**8.265**	**0.60**	**0.52**	**13.39**	**0.47**	**0.029394**	**0.043559**	**0.202351**	**0.379867**	**0.049950**	**0.353869**
**z15**	**186.856**	**0.088**	**186.378**	**0.651**	**181.465**	**8.588**	**0.35**	**0.50**	**6.62**	**0.19**	**0.029396**	**0.047732**	**0.201480**	**0.382329**	**0.049732**	**0.367130**
**z16**	**186.798**	**0.080**	**186.353**	**0.569**	**181.840**	**7.225**	**0.60**	**0.58**	**7.74**	**0.19**	**0.029387**	**0.043083**	**0.201450**	**0.334190**	**0.049740**	**0.308378**
**Rb_50**															
**z2**	**186.730**	**0.095**	**186.476**	**0.396**	**184.465**	**4.611**	**0.73**	**0.36**	**16.76**	**0.17**	**0.029375**	**0.051574**	**0.201597**	**0.232239**	**0.049797**	**0.195352**
**z4**	**186.827**	**0.208**	**187.074**	**2.295**	**191.354**	**29.394**	**0.72**	**0.46**	**6.77**	**0.89**	**0.029391**	**0.113170**	**0.202304**	**1.343141**	**0.049944**	**1.263345**
z5	186.882	0.078	187.020	0.246	189.929	3.003	0.49	0.45	13.43	0.11	0.029400	0.041908	0.202240	0.144183	0.049914	0.124967
**z6**	**186.668**	**0.085**	**186.312**	**0.489**	**182.986**	**6.278**	**0.45**	**0.40**	**6.74**	**0.16**	**0.029365**	**0.046184**	**0.201402**	**0.287164**	**0.049765**	**0.267535**
z9	186.944	0.227	186.390	2.232	180.562	28.555	0.72	0.41	6.85	0.88	0.029409	0.123444	0.201495	1.310587	0.049713	1.224822
z10	186.995	0.113	186.603	1.040	182.833	14.071	0.15	0.40	7.88	0.45	0.029417	0.061314	0.201747	0.610274	0.049762	0.603162
**Rb_22**															
z2	187.121	0.190	185.266	1.643	162.840	21.110	0.68	0.49	8.31	0.71	0.029438	0.102925	0.200165	0.969979	0.049337	0.902275
z4	187.311	0.171	186.706	1.265	180.107	15.650	0.79	0.79	11.73	0.69	0.029470	0.092412	0.201869	0.741825	0.049704	0.670669
z7	187.322	0.162	187.580	1.237	191.950	15.567	0.64	0.56	11.93	0.80	0.029470	0.087899	0.202904	0.722194	0.049957	0.668579
**z8**	**186.970**	**0.122**	**285.698**	**0.858**	**1199.872**	**6.004**	**0.67**	**0.89**	**11.69**	**0.47**	**0.029416**	**0.065646**	**0.324943**	**0.344619**	**0.080153**	**0.302844**
**z9**	**186.941**	**0.176**	**184.938**	**1.273**	**160.571**	**16.060**	**0.73**	**0.53**	**16.71**	**1.09**	**0.029409**	**0.095581**	**0.199777**	**0.753195**	**0.049289**	**0.685809**
**z11**	**186.948**	**0.123**	**186.012**	**1.035**	**175.272**	**13.071**	**0.76**	**0.57**	**4.56**	**0.21**	**0.029411**	**0.066383**	**0.201048**	**0.608893**	**0.049600**	**0.559378**
z13	186.647	0.112	185.262	0.743	168.806	9.249	0.73	0.47	6.04	0.18	0.029362	0.060847	0.200160	0.438566	0.049463	0.394677
**z14**	**186.959**	**0.446**	**186.223**	**5.020**	**178.052**	**64.641**	**0.75**	**0.50**	**3.64**	**1.03**	**0.029412**	**0.242434**	**0.201297**	**2.950206**	**0.049660**	**2.772187**

Highlighted grains were used in the weighted mean plot in FS. 3
a Corrected for initial Th/U disequilibrium using radiogenic 208Pb and Th/U[magma] = 3.50000.
b Isotopic dates calculated using λ238 = 1.55125E-10 [22] and λ235 = 9.8485E-10 [22]
c Th contents calculated from radiogenic 208Pb and 230Th-corrected 206Pb/238U date of the sample, assuming concordance between U-Pb Th-Pb systems.
d Total mass of radiogenic Pb.
e Total mass of common Pb.
f Measured ratios corrected for fractionation, tracer and blank.

Age-depth model data table TS.2

Str. Height (cm)	Age (Ma)	2σ (Ma)	Str. Height (cm)	Age (Ma)	2σ (Ma)	Str. Height (cm)	Age (Ma)	2σ (Ma)	Str. Height (cm)	Age (Ma)	2σ (Ma)	Str. Height (cm)	Age (Ma)	2σ (Ma)
8500	184.02	0.10	8150	184.11	0.23	7800	184.19	0.25	7450	184.28	0.23	7100	184.42	0.094
8490	184.03	0.11	8140	184.11	0.23	7790	184.20	0.25	7440	184.28	0.23	7090	184.42	0.094
8480	184.03	0.12	8130	184.12	0.23	7780	184.20	0.25	7430	184.28	0.23	7080	184.42	0.094
8470	184.03	0.14	8120	184.12	0.23	7770	184.20	0.25	7420	184.29	0.23	7070	184.42	0.094
8460	184.04	0.14	8110	184.12	0.23	7760	184.20	0.25	7410	184.29	0.23	7060	184.42	0.094
8450	184.04	0.15	8100	184.12	0.23	7750	184.21	0.25	7400	184.29	0.22	7050	184.42	0.094
8440	184.04	0.15	8090	184.13	0.24	7740	184.21	0.25	7390	184.29	0.22	7040	184.42	0.094
8430	184.04	0.16	8080	184.13	0.24	7730	184.21	0.25	7380	184.30	0.22	7030	184.43	0.094
8420	184.05	0.17	8070	184.13	0.24	7720	184.21	0.25	7370	184.30	0.22	7020	184.43	0.094
8410	184.05	0.17	8060	184.13	0.24	7710	184.22	0.25	7360	184.30	0.22	7010	184.43	0.094
8400	184.05	0.17	8050	184.14	0.24	7700	184.22	0.25	7350	184.30	0.22	7000	184.43	0.094
8390	184.05	0.17	8040	184.14	0.24	7690	184.22	0.25	7340	184.31	0.22	6990	184.43	0.094
8380	184.06	0.18	8030	184.14	0.24	7680	184.22	0.25	7330	184.31	0.22	6980	184.43	0.094
8370	184.06	0.18	8020	184.14	0.24	7670	184.23	0.25	7320	184.31	0.22	6970	184.43	0.094
8360	184.06	0.19	8010	184.14	0.25	7660	184.23	0.25	7310	184.31	0.22	6960	184.43	0.093
8350	184.06	0.19	8000	184.15	0.25	7650	184.23	0.25	7300	184.32	0.22	6950	184.43	0.094
8340	184.07	0.20	7990	184.15	0.25	7640	184.23	0.25	7290	184.32	0.22	6940	184.43	0.093
8330	184.07	0.20	7980	184.15	0.25	7630	184.24	0.25	7280	184.32	0.21	6930	184.44	0.094
8320	184.07	0.20	7970	184.15	0.24	7620	184.24	0.25	7270	184.32	0.21	6920	184.44	0.094
8310	184.07	0.20	7960	184.16	0.24	7610	184.24	0.25	7260	184.33	0.21	6910	184.44	0.094
8300	184.08	0.21	7950	184.16	0.24	7600	184.24	0.24	7250	184.33	0.21	6900	184.44	0.094
8290	184.08	0.21	7940	184.16	0.25	7590	184.25	0.24	7240	184.33	0.20	6890	184.44	0.094
8280	184.08	0.21	7930	184.16	0.25	7580	184.25	0.24	7230	184.33	0.20	6880	184.44	0.094
8270	184.08	0.21	7920	184.17	0.25	7570	184.25	0.24	7220	184.33	0.19	6870	184.44	0.093
8260	184.08	0.21	7910	184.17	0.25	7560	184.25	0.24	7210	184.34	0.19	6860	184.44	0.093

8250	184.09	0.21	7900	184.17	0.25	7550	184.26	0.24	7200	184.34	0.18	6850	184.44	0.093
8240	184.09	0.22	7890	184.17	0.25	7540	184.26	0.24	7190	184.34	0.18	6840	184.44	0.093
8230	184.09	0.22	7880	184.18	0.25	7530	184.26	0.24	7180	184.35	0.17	6830	184.44	0.093
8220	184.09	0.22	7870	184.18	0.25	7520	184.26	0.24	7170	184.35	0.16	6820	184.45	0.093
8210	184.10	0.22	7860	184.18	0.25	7510	184.26	0.24	7160	184.35	0.14	6810	184.45	0.094
8200	184.10	0.22	7850	184.18	0.25	7500	184.27	0.24	7150	184.36	0.13	6800	184.45	0.093
8190	184.10	0.23	7840	184.18	0.25	7490	184.27	0.24	7140	184.36	0.13	6790	184.45	0.093
8180	184.10	0.23	7830	184.19	0.25	7480	184.27	0.24	7130	184.42	0.10	6780	184.45	0.093
8170	184.11	0.23	7820	184.19	0.24	7470	184.27	0.23	7120	184.42	0.09	6770	184.45	0.094
8160	184.11	0.23	7810	184.19	0.24	7460	184.28	0.23	7110	184.42	0.09	6760	184.45	0.092
												6750	184.45	0.092

6740	184.453	0.092	6390	184.490	0.090	6040	184.526	0.088	5690	184.63	0.17	5340	184.84	0.24
6730	184.454	0.091	6380	184.490	0.091	6030	184.527	0.088	5680	184.63	0.17	5330	184.84	0.24
6720	184.455	0.091	6370	184.491	0.091	6020	184.528	0.088	5670	184.64	0.18	5320	184.85	0.24
6710	184.456	0.091	6360	184.493	0.091	6010	184.529	0.088	5660	184.65	0.18	5310	184.86	0.24
6700	184.457	0.091	6350	184.494	0.089	6000	184.530	0.089	5650	184.65	0.18	5300	184.86	0.24
6690	184.458	0.090	6340	184.495	0.090	5990	184.531	0.089	5640	184.66	0.19	5290	184.87	0.24
6680	184.459	0.090	6330	184.496	0.090	5980	184.532	0.090	5630	184.66	0.19	5280	184.87	0.24
6670	184.461	0.090	6320	184.497	0.091	5970	184.533	0.090	5620	184.67	0.19	5270	184.88	0.24
6660	184.461	0.090	6310	184.498	0.091	5960	184.535	0.090	5610	184.68	0.19	5260	184.89	0.24
6650	184.462	0.090	6300	184.499	0.091	5950	184.536	0.091	5600	184.68	0.20	5250	184.89	0.24
6640	184.463	0.090	6290	184.500	0.091	5940	184.537	0.092	5590	184.69	0.20	5240	184.90	0.24
6630	184.465	0.089	6280	184.500	0.091	5930	184.538	0.087	5580	184.69	0.21	5230	184.90	0.24
6620	184.466	0.089	6270	184.502	0.091	5920	184.539	0.085	5570	184.70	0.21	5220	184.91	0.24
6610	184.467	0.089	6260	184.503	0.092	5910	184.540	0.083	5560	184.71	0.21	5210	184.91	0.24
6600	184.468	0.089	6250	184.504	0.092	5900	184.542	0.082	5550	184.71	0.21	5200	184.92	0.24
6590	184.469	0.089	6240	184.505	0.092	5890	184.543	0.079	5540	184.72	0.22	5190	184.93	0.24
6580	184.470	0.089	6230	184.506	0.092	5880	184.544	0.075	5530	184.72	0.22	5180	184.93	0.24
6570	184.472	0.089	6220	184.507	0.091	5870	184.546	0.073	5520	184.73	0.22	5170	184.94	0.24

6560	184.473	0.089	6210	184.508	0.091	5860	184.547	0.071	5510	184.74	0.22	5160	184.95	0.24
6550	184.474	0.089	6200	184.509	0.091	5850	184.548	0.070	5500	184.74	0.23	5150	184.95	0.24
6540	184.475	0.089	6190	184.510	0.091	5840	184.549	0.070	5490	184.75	0.23	5140	184.96	0.24
6530	184.476	0.090	6180	184.511	0.091	5830	184.551	0.070	5480	184.75	0.23	5130	184.96	0.24
6520	184.476	0.090	6170	184.512	0.091	5820	184.553	0.067	5470	184.76	0.23	5120	184.97	0.24
6510	184.477	0.090	6160	184.513	0.091	5810	184.554	0.062	5460	184.77	0.23	5110	184.97	0.24
6500	184.478	0.090	6150	184.514	0.091	5800	184.557	0.053	5450	184.77	0.23	5100	184.98	0.24
6490	184.479	0.090	6140	184.515	0.091	5790	184.565	0.092	5440	184.78	0.23	5090	184.99	0.24
6480	184.480	0.091	6130	184.516	0.091	5780	184.57	0.12	5430	184.78	0.23	5080	184.99	0.24
6470	184.481	0.091	6120	184.517	0.091	5770	184.58	0.14	5420	184.79	0.23	5070	185.00	0.24
6460	184.482	0.091	6110	184.518	0.091	5760	184.58	0.14	5410	184.80	0.23	5060	185.00	0.24
6450	184.483	0.090	6100	184.519	0.091	5750	184.59	0.15	5400	184.80	0.23	5050	185.01	0.24
6440	184.484	0.090	6090	184.520	0.089	5740	184.60	0.16	5390	184.81	0.23	5040	185.02	0.24
6430	184.485	0.090	6080	184.521	0.088	5730	184.60	0.16	5380	184.81	0.23	5030	185.02	0.24
6420	184.486	0.090	6070	184.523	0.088	5720	184.61	0.17	5370	184.82	0.23	5020	185.03	0.25
6410	184.487	0.090	6060	184.524	0.087	5710	184.61	0.17	5360	184.83	0.23	5010	185.03	0.25
6400	184.489	0.090	6050	184.525	0.088	5700	184.62	0.17	5350	184.83	0.24	5000	185.04	0.25
												4990	185.04	0.25

4980	185.05	0.25	4630	185.21	0.12	4280	185.27	0.14	3930	185.34	0.14	3580	185.40	0.13
4970	185.06	0.25	4620	185.21	0.12	4270	185.27	0.14	3920	185.34	0.14	3570	185.40	0.13
4960	185.06	0.25	4610	185.21	0.12	4260	185.27	0.14	3910	185.34	0.14	3560	185.40	0.13
4950	185.07	0.25	4600	185.21	0.12	4250	185.28	0.14	3900	185.34	0.14	3550	185.41	0.13
4940	185.07	0.25	4590	185.21	0.12	4240	185.28	0.14	3890	185.34	0.14	3540	185.41	0.13
4930	185.08	0.25	4580	185.22	0.12	4230	185.28	0.14	3880	185.35	0.14	3530	185.41	0.13
4920	185.08	0.25	4570	185.22	0.12	4220	185.28	0.14	3870	185.35	0.14	3520	185.41	0.13
4910	185.09	0.25	4560	185.22	0.13	4210	185.28	0.14	3860	185.35	0.14	3510	185.41	0.13
4900	185.10	0.25	4550	185.22	0.13	4200	185.28	0.14	3850	185.35	0.14	3500	185.42	0.13
4890	185.10	0.24	4540	185.22	0.13	4190	185.29	0.14	3840	185.35	0.14	3490	185.42	0.13
4880	185.11	0.23	4530	185.23	0.13	4180	185.29	0.14	3830	185.35	0.14	3480	185.42	0.13

4870	185.11	0.23	4520	185.23	0.13	4170	185.29	0.14	3820	185.36	0.14	3470	185.42	0.13
4860	185.12	0.22	4510	185.23	0.13	4160	185.29	0.14	3810	185.36	0.14	3460	185.42	0.13
4850	185.13	0.23	4500	185.23	0.13	4150	185.29	0.14	3800	185.36	0.14	3450	185.42	0.13
4840	185.13	0.22	4490	185.23	0.13	4140	185.30	0.14	3790	185.36	0.14	3440	185.43	0.13
4830	185.14	0.22	4480	185.23	0.13	4130	185.30	0.14	3780	185.36	0.14	3430	185.43	0.13
4820	185.14	0.22	4470	185.24	0.13	4120	185.30	0.14	3770	185.37	0.14	3420	185.43	0.13
4810	185.15	0.20	4460	185.24	0.14	4110	185.30	0.14	3760	185.37	0.14	3410	185.43	0.13
4800	185.16	0.19	4450	185.24	0.14	4100	185.30	0.14	3750	185.37	0.14	3400	185.43	0.13
4790	185.16	0.13	4440	185.24	0.14	4090	185.31	0.14	3740	185.37	0.14	3390	185.44	0.13
4780	185.170	0.080	4430	185.24	0.14	4080	185.31	0.14	3730	185.37	0.13	3380	185.44	0.13
4770	185.174	0.080	4420	185.25	0.14	4070	185.31	0.15	3720	185.37	0.13	3370	185.44	0.13
4760	185.177	0.084	4410	185.25	0.14	4060	185.31	0.15	3710	185.38	0.13	3360	185.44	0.12
4750	185.180	0.087	4400	185.25	0.14	4050	185.31	0.15	3700	185.38	0.13	3350	185.44	0.12
4740	185.183	0.088	4390	185.25	0.14	4040	185.31	0.15	3690	185.38	0.13	3340	185.44	0.12
4730	185.186	0.091	4380	185.25	0.14	4030	185.32	0.15	3680	185.38	0.13	3330	185.45	0.12
4720	185.189	0.092	4370	185.25	0.14	4020	185.32	0.15	3670	185.38	0.13	3320	185.45	0.12
4710	185.191	0.095	4360	185.26	0.14	4010	185.32	0.15	3660	185.39	0.13	3310	185.45	0.11
4700	185.193	0.098	4350	185.26	0.14	4000	185.32	0.15	3650	185.39	0.13	3300	185.45	0.11
4690	185.20	0.10	4340	185.26	0.14	3990	185.32	0.15	3640	185.39	0.13	3290	185.45	0.11
4680	185.20	0.10	4330	185.26	0.14	3980	185.33	0.15	3630	185.39	0.13	3280	185.46	0.11
4670	185.20	0.11	4320	185.26	0.14	3970	185.33	0.15	3620	185.39	0.13	3270	185.46	0.11
4660	185.20	0.11	4310	185.27	0.14	3960	185.33	0.15	3610	185.39	0.13	3260	185.46	0.10
4650	185.20	0.11	4300	185.27	0.14	3950	185.33	0.14	3600	185.40	0.13	3250	185.46	0.10
4640	185.21	0.11	4290	185.27	0.14	3940	185.33	0.14	3590	185.40	0.13	3240	185.46	0.10
												3230	185.47	0.10

3220	185.468	0.098	2870	185.66	0.33	2520	185.86	0.39	2170	186.05	0.41	1820	186.25	0.41
3210	185.471	0.088	2860	185.67	0.34	2510	185.86	0.39	2160	186.06	0.41	1810	186.25	0.41
3200	185.474	0.051	2850	185.67	0.34	2500	185.87	0.39	2150	186.06	0.41	1800	186.26	0.41

3190	185.48	0.10	2840	185.68	0.34	2490	185.87	0.39	2140	186.07	0.41	1790	186.26	0.41
3180	185.49	0.14	2830	185.69	0.35	2480	185.88	0.39	2130	186.07	0.41	1780	186.27	0.41
3170	185.50	0.16	2820	185.69	0.35	2470	185.88	0.39	2120	186.08	0.42	1770	186.27	0.41
3160	185.50	0.18	2810	185.70	0.35	2460	185.89	0.39	2110	186.09	0.42	1760	186.28	0.41
3150	185.51	0.19	2800	185.70	0.35	2450	185.90	0.39	2100	186.09	0.42	1750	186.28	0.41
3140	185.51	0.20	2790	185.71	0.35	2440	185.90	0.39	2090	186.10	0.42	1740	186.29	0.41
3130	185.52	0.22	2780	185.71	0.36	2430	185.91	0.39	2080	186.10	0.42	1730	186.30	0.41
3120	185.52	0.24	2770	185.72	0.36	2420	185.91	0.39	2070	186.11	0.42	1720	186.30	0.41
3110	185.53	0.24	2760	185.72	0.36	2410	185.92	0.39	2060	186.11	0.41	1710	186.31	0.41
3100	185.53	0.25	2750	185.73	0.36	2400	185.92	0.40	2050	186.12	0.41	1700	186.31	0.41
3090	185.54	0.26	2740	185.74	0.37	2390	185.93	0.40	2040	186.12	0.41	1690	186.32	0.41
3080	185.55	0.28	2730	185.74	0.37	2380	185.93	0.40	2030	186.13	0.41	1680	186.32	0.41
3070	185.55	0.30	2720	185.75	0.37	2370	185.94	0.40	2020	186.13	0.42	1670	186.33	0.41
3060	185.56	0.30	2710	185.75	0.37	2360	185.95	0.40	2010	186.14	0.42	1660	186.33	0.41
3050	185.56	0.30	2700	185.76	0.37	2350	185.95	0.40	2000	186.14	0.42	1650	186.34	0.41
3040	185.57	0.30	2690	185.76	0.37	2340	185.96	0.40	1990	186.15	0.42	1640	186.34	0.41
3030	185.57	0.31	2680	185.77	0.37	2330	185.96	0.40	1980	186.16	0.42	1630	186.35	0.41
3020	185.58	0.31	2670	185.77	0.37	2320	185.97	0.40	1970	186.16	0.42	1620	186.35	0.40
3010	185.58	0.31	2660	185.78	0.37	2310	185.97	0.40	1960	186.17	0.42	1610	186.36	0.39
3000	185.59	0.32	2650	185.79	0.37	2300	185.98	0.40	1950	186.17	0.42	1600	186.36	0.38
2990	185.59	0.32	2640	185.79	0.38	2290	185.98	0.40	1940	186.18	0.42	1590	186.37	0.38
2980	185.60	0.32	2630	185.80	0.38	2280	185.99	0.40	1930	186.18	0.42	1580	186.38	0.38
2970	185.61	0.32	2620	185.80	0.38	2270	186.00	0.40	1920	186.19	0.42	1570	186.38	0.36
2960	185.61	0.33	2610	185.81	0.38	2260	186.00	0.40	1910	186.19	0.42	1560	186.39	0.36
2950	185.62	0.33	2600	185.81	0.38	2250	186.01	0.40	1900	186.20	0.42	1550	186.39	0.36
2940	185.62	0.33	2590	185.82	0.38	2240	186.01	0.41	1890	186.21	0.42	1540	186.40	0.36
2930	185.63	0.33	2580	185.82	0.38	2230	186.02	0.41	1880	186.21	0.42	1530	186.40	0.34
2920	185.63	0.33	2570	185.83	0.38	2220	186.02	0.41	1870	186.22	0.42	1520	186.41	0.34
2910	185.64	0.33	2560	185.84	0.38	2210	186.03	0.41	1860	186.22	0.42	1510	186.42	0.34
2900	185.65	0.33	2550	185.84	0.38	2200	186.03	0.41	1850	186.23	0.42	1500	186.42	0.34
2890	185.65	0.33	2540	185.85	0.38	2190	186.04	0.41	1840	186.23	0.42	1490	186.43	0.33

2880	185.66	0.33	2530	185.85	0.39	2180	186.05	0.41	1830	186.24	0.42	1480	186.44	0.32
												1470	186.44	0.31
1460	186.45	0.30	1110	186.59	0.13	760	186.702	0.068	410	186.760	0.059	60	186.819	0.064
1450	186.46	0.30	1100	186.59	0.13	750	186.704	0.067	400	186.762	0.059	50	186.821	0.063
1440	186.47	0.27	1090	186.60	0.13	740	186.707	0.067	390	186.763	0.059	40	186.822	0.063
1430	186.47	0.28	1080	186.60	0.13	730	186.708	0.067	380	186.765	0.059	30	186.824	0.052
1420	186.49	0.17	1070	186.60	0.13	720	186.710	0.067	370	186.767	0.059	20	186.826	0.048
1410	186.49	0.17	1060	186.61	0.13	710	186.712	0.067	360	186.768	0.059	10	186.830	0.043
1400	186.50	0.17	1050	186.61	0.13	700	186.714	0.067	350	186.770	0.059	0	186.839	0.043
1390	186.50	0.17	1040	186.61	0.13	690	186.716	0.066	340	186.771	0.059	-10	186.848	0.044
1380	186.51	0.17	1030	186.62	0.13	680	186.718	0.066	330	186.773	0.059	-20	186.857	0.046
1370	186.51	0.17	1020	186.62	0.13	670	186.719	0.066	320	186.775	0.059	-30	186.867	0.048
1360	186.51	0.16	1010	186.62	0.13	660	186.721	0.065	310	186.776	0.059	-40	186.876	0.050
1350	186.52	0.16	1000	186.62	0.13	650	186.723	0.064	300	186.778	0.059	-50	186.886	0.052
1340	186.52	0.16	990	186.63	0.12	640	186.724	0.064	290	186.780	0.060	-60	186.895	0.054
1330	186.52	0.16	980	186.63	0.12	630	186.725	0.065	280	186.781	0.060	-70	186.904	0.056
1320	186.53	0.16	970	186.63	0.12	620	186.727	0.064	270	186.783	0.059	-80	186.913	0.059
1310	186.53	0.15	960	186.64	0.12	610	186.729	0.064	260	186.785	0.059	-90	186.923	0.062
1300	186.53	0.16	950	186.64	0.12	600	186.730	0.064	250	186.786	0.059	-100	186.932	0.066
1290	186.53	0.15	940	186.64	0.12	590	186.732	0.063	240	186.788	0.059	-110	186.940	0.069
1280	186.54	0.15	930	186.65	0.12	580	186.734	0.062	230	186.789	0.059	-120	186.949	0.074
1270	186.54	0.15	920	186.65	0.12	570	186.735	0.061	220	186.791	0.060	-130	186.958	0.078
1260	186.54	0.15	910	186.65	0.12	560	186.736	0.060	210	186.793	0.061	-140	186.968	0.078
1250	186.55	0.15	900	186.65	0.12	550	186.738	0.060	200	186.794	0.061	-150	186.979	0.082
1240	186.55	0.15	890	186.66	0.12	540	186.740	0.060	190	186.796	0.061	-160	186.985	0.090
1230	186.55	0.14	880	186.66	0.11	530	186.741	0.059	180	186.798	0.061	-170	186.99	0.10
1220	186.56	0.14	870	186.66	0.11	520	186.743	0.059	170	186.799	0.061	-180	186.99	0.12
1210	186.56	0.14	860	186.67	0.10	510	186.744	0.059	160	186.801	0.061	-190	186.99	0.13
1200	186.56	0.14	850	186.67	0.10	500	186.746	0.059	150	186.803	0.061	-200	187.00	0.14

1190	186.57	0.14	840	186.67	0.10	490	186.747	0.059	140	186.805	0.061
1180	186.57	0.14	830	186.68	0.10	480	186.749	0.059	130	186.807	0.062
1170	186.57	0.14	820	186.68	0.10	470	186.751	0.059	120	186.809	0.062
1160	186.58	0.14	810	186.685	0.097	460	186.752	0.058	110	186.811	0.063
1150	186.58	0.14	800	186.688	0.098	450	186.754	0.058	100	186.812	0.063
1140	186.58	0.13	790	186.692	0.097	440	186.755	0.059	90	186.814	0.063
1130	186.58	0.13	780	186.695	0.099	430	186.757	0.059	80	186.815	0.063
1120	186.59	0.13	770	186.699	0.067	420	186.759	0.059	70	186.817	0.063

Geochemical data TS.3

		Rock Eval					Organic C Isotopes		Mercury Data	
Str. Height (m)	Sample Name	Tmax (oC)	HI (mg HC/gTOC)	OI (mg CO2/gTOC)	TOC (%)	S2 (mgHC/g)	TOC (%)	d13C ‰ (VPDB)	Hg (ppm)	Hg/TOC (ppm/%)
81.00	SC2.1	450.00	62.00	16.00	1.11	0.69	1.03	-30.30	0.45	0.40
80.70	SC2.2	448.00	110.00	9.00	1.68	1.84	1.61	-30.30	0.45	0.27
80.00	SC4.1	448.00	115.00	10.00	2.13	2.45	2.25	-29.70	0.49	0.23
79.66	SC4.2	445.00	79.00	14.00	1.90	1.51	1.84	-29.70	0.38	0.20
79.32	SC4.3	444.00	114.00	75.00	0.44	0.50	0.56	-30.90	0.11	0.25
78.98	SC4.4	NA	NA	NA	NA	NA	0.20	-30.70	0.45	NA
78.64	SC4.5	443.00	59.00	25.00	1.26	0.74	1.21	-30.50	0.26	0.20
75.68	WS16.5	450.70	60.33	72.08	2.59	1.56	3.79	-29.17	0.15	0.06
74.86	WS16.4	447.60	51.17	114.50	1.64	0.84	2.49	-30.04	0.11	0.07
74.24	WS16.3	448.80	80.90	61.57	2.05	1.66	2.87	-28.91	0.09	0.04
73.62	WS16.2	NA	NA	NA	NA	NA	3.31	-29.09	0.08	NA
73.00	WS16.1	446.33	73.08	60.21	2.08	1.52	2.68	-28.70	0.07	0.03
72.50	WS14.4	460.40	59.67	86.07	0.93	0.56	1.25	-29.71	0.10	0.11
72.10	WS14.3	NA	NA	NA	NA	NA	3.30	-29.41	0.07	NA
71.85	SC8.1	454.00	54.00	34.00	1.12	0.61	1.17	-29.60	0.23	0.21
71.70	WS14.2	451.50	53.81	76.43	2.27	1.22	2.68	-29.42	0.10	0.05
71.37	SC8.2	460.00	64.00	52.00	0.91	0.58	1.03	-29.50	0.18	0.20
71.30	WS12.3	444.67	72.75	70.03	2.80	2.03	5.34	-28.96	0.07	0.02
70.89	SC8.3	NA	NA	NA	NA	NA	1.15	-30.10	0.20	NA
70.80	WS12.2	NA	NA	NA	NA	NA	6.01	-29.00	0.05	NA
70.41	SC8.4	NA	NA	NA	NA	NA	0.23	-30.80	0.19	NA
70.30	WS12.1	456.00	56.18	70.60	1.37	0.77	2.13	-29.91	0.08	0.06
69.93	SC8.5	459.00	96.00	27.00	1.28	1.23	1.23	-29.60	0.19	0.15
68.50	WS8	448.90	57.85	71.71	2.21	1.28	3.38	-29.44	0.05	0.02
68.50	WS6.5	NA	NA	NA	NA	NA	3.33	-29.46	0.05	NA
68.20	WS6.4	NA	NA	NA	NA	NA	3.19	-29.32	0.06	NA
67.80	WS6.3	444.04	73.53	64.10	1.07	0.78	4.41	-28.27	0.08	0.08
67.40	WS6.2	444.33	43.30	94.24	3.38	1.46	4.08	-28.49	0.10	0.03
67.10	WS6.1	NA	NA	NA	NA	NA	3.30	-29.26	0.10	NA
66.00	WS3	456.67	97.71	33.04	0.93	0.91	6.68	-28.85	0.01	0.02
61.50	SC10.1	446.00	177.00	14.00	3.46	6.13	4.39	-26.90	0.04	0.01
61.00	SC10.2	454.00	47.00	87.00	1.77	0.83	2.56	-28.39	0.05	0.03

60.75	SC11.C1	456	111.46	34	1.02	1.14	7.33	-29.53	NA	NA
60.55	SC11.02	452.00	38.00	129.00	1.29	0.49	4.79	-29.50	0.15	0.12
59.79	SC11.03	453.00	45.00	101.00	1.34	0.60	2.23	-28.84	0.16	0.12
59.41	SC11.04	457.00	36.00	121.00	1.46	0.52	2.92	-28.85	0.29	0.20
59.03	SC11.05	449.65	38.07	100.46	1.87	0.71	2.37	-28.80	0.41	0.22
58.65	SC11.06	456.00	47.00	89.00	1.84	0.86	2.32	-29.50	0.48	0.26
58.28	SC11.07	NA	NA	NA	NA	NA	2.58	-28.29	0.49	NA
57.90	SC11.08	454.00	37.00	93.00	1.77	0.66	3.10	-28.97	0.35	0.20
57.52	SC11.09	451.00	54.00	84.00	1.77	0.95	2.72	-29.06	0.41	0.23
58.00	SC11.C2	451.00	38.00	128.00	1.85	0.70	2.76	-29.62	NA	NA
57.14	SC11.10	NA	NA	NA	NA	NA	2.83	-29.11	0.41	NA
56.76	SC11.11	451.00	49.00	98.00	1.06	0.52	2.83	-28.94	0.51	0.48
56.38	SC11.12	455.00	40.00	95.00	1.66	0.66	2.40	-30.00	0.27	0.17
54.45	SC13.1	NA	NA	NA	NA	NA	1.34	-30.01	0.05	NA
54.30	SC13.2	454.00	38.00	110.00	1.18	0.44	1.36	-29.63	0.04	0.03
54.15	SC13.3	453.74	37.16	113.20	1.21	0.45	1.31	-29.65	0.04	0.03
53.80	SC15.1	NA	NA	NA	NA	NA	1.93	-28.97	0.05	NA
53.51	SC15.2	450.00	40.00	98.00	1.18	0.48	2.43	-28.51	0.05	0.04
53.23	SC15.3	NA	NA	NA	NA	NA	1.35	-29.94	0.03	NA
53.00	SC15.C	456.00	179.00	9.00	2.62	4.71	2.78	-29.63	NA	NA
52.94	SC15.4	458.30	30.53	95.90	1.61	0.49	1.96	-29.21	0.04	0.03
51.60	SC17.1	452.30	52.36	101.68	1.08	0.57	1.38	-29.36	0.03	0.03
51.30	SC17.C1	462.00	127.00	32.00	0.85	1.08	2.56	-29.38	NA	NA
51.40	SC17.2	NA	NA	NA	NA	NA	0.44	-27.60	0.07	NA
51.20	SC17.3	469.26	76.67	125.23	0.40	0.31	1.29	-29.40	0.06	0.14
51.00	SC17.4	457.00	32.00	310.00	0.97	0.31	0.96	-29.01	0.08	0.08
50.80	SC17.5	NA	NA	NA	NA	NA	0.74	-29.41	0.14	NA
50.60	SC17.6	NA	NA	NA	NA	NA	1.87	-27.99	0.05	NA
50.40	SC17.7	453.26	170.88	225.63	0.10	0.18	0.13	-29.01	0.03	0.26
50.20	SC17.8	NA	NA	NA	NA	NA	0.65	-28.91	0.04	0.06
50.10	SC17.C2	454.00	76.00	204.00	0.22	0.17	0.25	-28.92	NA	NA
50.00	SC17.9	NA	NA	NA	NA	NA	3.49	-28.57	0.01	NA
48.90	SC19.1	449.48	57.37	64.16	1.43	0.82	1.44	-28.79	0.04	0.03
48.49	SC19.2	NA	NA	NA	NA	NA	2.61	-28.54	0.04	NA
48.08	SC19.3	453.20	43.46	75.60	2.99	1.30	4.03	-28.70	0.08	0.03
47.85	SC19.C	461.00	60.00	62.00	1.80	1.09	1.74	-29.08	NA	NA
47.67	SC19.4	NA	NA	NA	NA	NA	3.83	-29.11	0.04	NA
47.26	SC19.5	457.52	33.97	97.43	1.60	0.54	2.17	-29.30	0.06	0.04
46.00	SC21.01	455.83	47.71	89.78	1.72	0.82	2.04	-29.20	0.10	0.06

45.85	SC21.C1	453.00	195.00	6.00	3.62	7.06	5.78	-29.12	NA	NA
45.62	SC21.02	NA	NA	NA	NA	NA	1.36	-28.88	0.13	NA
45.24	SC21.03	450.78	57.27	88.83	1.83	1.05	2.11	-28.53	0.04	0.02
44.48	SC21.05	NA	NA	NA	NA	NA	3.49	-28.40	0.04	NA
44.30	SC21.C2	449.00	71.00	87.00	2.57	1.82	4.08	-29.21	NA	NA
44.10	SC21.06	NA	NA	NA	NA	NA	1.70	-29.60	0.05	NA
43.72	SC21.07	450.13	40.42	79.87	2.00	0.81	1.96	-28.39	0.04	0.02
43.34	SC21.08	NA	NA	NA	NA	NA	1.75	-28.85	0.04	NA
42.96	SC21.09	449.87	155.67	11.91	4.49	6.98	2.23	-28.85	0.06	0.01
42.70	SC21.C3	455.00	213.00	8.00	3.34	7.11	5.17	-29.94	NA	NA
42.58	SC21.10	NA	NA	NA	NA	NA	2.02	-28.80	0.04	NA
42.20	SC21.11	445.70	40.13	104.30	1.20	0.48	2.59	-29.00	0.04	0.03
41.83	SC21.12	NA	NA	NA	NA	NA	2.43	-28.29	0.03	NA
41.45	SC21.13	450.90	67.57	79.70	2.06	1.39	3.08	-28.98	0.04	0.02
41.07	SC21.14	NA	NA	NA	NA	NA	2.95	-29.12	0.03	NA
40.90	SC21.C4	454.00	228.00	9.00	3.69	8.40	8.17	-29.80	NA	NA
40.69	SC21.15	445.60	140.19	27.27	2.63	3.69	4.53	-29.20	0.03	0.01
40.31	SC21.16	NA	NA	NA	NA	NA	2.82	-28.94	0.05	NA
39.93	SC21.17	448.00	163.69	17.36	2.76	4.53	2.18	-29.39	0.03	0.01
39.55	SC21.18	NA	NA	NA	NA	NA	1.13	-29.37	0.06	NA
39.17	SC21.19	448.17	51.34	80.53	1.93	0.99	8.55	-30.93	0.06	0.03
38.79	SC21.2	NA	NA	NA	NA	NA	0.94	-30.02	0.08	NA
38.41	SC21.21	448.22	37.88	112.58	1.55	0.59	1.94	-30.20	0.06	0.04
38.03	SC21.22	NA	NA	NA	NA	NA	1.16	-29.64	0.05	NA
37.45	SC23	452.70	40.58	100.61	1.32	0.53	1.76	-29.66	0.06	0.05
36.40	SC25.1	NA	NA	NA	NA	NA	1.84	-29.20	0.05	NA
35.76	SC25.2	452.61	30.94	103.35	1.32	0.41	2.53	-28.98	0.04	0.03
34.48	SC25.4	451.00	46.00	87.00	1.70	0.78	1.92	-29.20	0.05	0.03
33.84	SC25.6	449.00	58.17	76.02	1.55	0.90	2.35	-29.30	0.03	0.02
33.00	SC27.1	NA	NA	NA	NA	NA	1.64	-29.30	0.04	NA
32.90	SC27.C	455.00	123.00	40.00	1.98	2.44	2.82	-29.50	NA	NA
32.73	SC27.2	NA	NA	NA	NA	NA	2.03	-29.23	0.05	NA
32.45	SC27.3	NA	NA	NA	NA	NA	2.02	-29.00	0.06	NA
32.00	SC29.1	NA	NA	NA	NA	NA	1.87	-29.10	0.05	NA
31.64	SC29.2	450.00	44.00	133.00	1.04	0.46	1.55	-29.00	0.03	0.03
31.28	SC29.4	455.50	35.02	83.76	1.70	0.59	1.70	-29.20	0.04	0.02
30.92	SC29.5	NA	NA	NA	NA	NA	3.29	-28.52	0.03	NA
30.56	SC29.7	449.30	28.32	103.68	1.28	0.36	1.60	-29.40	0.03	0.03
30.00	SC31.1	NA	NA	NA	NA	NA	1.48	-29.40	0.03	NA

29.65	SC31.2	452.35	56.86	67.82	1.86	1.06	1.92	-28.80	0.03	0.02
29.30	SC32	445.96	115.53	67.56	0.53	0.61	2.52	-29.56	0.01	0.02
29.00	SC33.1	NA	NA	NA	NA	NA	1.73	-29.60	0.03	NA
28.43	SC33.2	NA	NA	NA	NA	NA	1.88	-29.30	0.05	NA
27.86	SC33.3	NA	NA	NA	NA	NA	1.69	-28.80	0.05	NA
27.29	SC33.4	448.10	56.66	86.92	1.68	0.95	2.63	-28.80	0.04	0.02
26.72	SC33.5	453.09	34.76	95.11	1.43	0.50	1.52	-29.20	0.04	0.03
26.00	SC35.1	451.80	41.71	85.19	1.81	0.76	1.81	-29.00	0.06	0.03
17.28	SC35.2	NA	NA	NA	NA	NA	1.67	-29.20	0.03	NA
8.57	SC35.3	453.00	36.00	107.00	1.64	0.60	1.83	-29.20	0.05	0.03
24.10	SC37	455.91	37.01	125.94	1.20	0.44	1.65	-28.50	0.04	0.03
23.35	SC39.1	463.00	61.00	107.00	0.61	0.37	1.54	-29.70	0.06	0.10
22.97	SC39.2	459.00	50.00	92.00	1.20	0.60	1.60	-29.50	0.18	0.15
22.58	SC39.3	448.00	46.00	124.00	0.54	0.25	2.14	-30.10	0.19	0.34
22.20	SC40	459.00	68.00	109.00	0.69	0.47	2.08	-29.50	0.14	0.21
18.90	SC42.1	461.00	41.00	155.00	0.56	0.23	1.04	-29.90	0.73	1.30
18.54	SC42.2	463.00	59.00	59.00	0.97	0.57	1.22	-30.00	0.90	0.92
18.17	SC42.3	460.00	43.00	114.00	0.72	0.31	0.88	-30.20	0.43	0.60
17.81	SC42.4	461.00	46.00	104.00	0.80	0.37	1.11	-30.00	0.92	1.15
17.44	SC42.5	462.00	37.00	180.00	0.49	0.18	0.71	-29.90	1.53	3.11
17.08	SC42.6	460.00	32.00	150.00	0.60	0.19	0.70	-30.10	1.93	3.21
16.71	SC42.7	462.00	39.00	126.00	0.57	0.22	0.67	-30.10	2.18	3.82
15.85	SC44.2	462.00	53.00	82.00	0.83	0.44	0.95	-30.40	1.08	1.30
15.35	SC46.1	462.00	47.00	111.00	0.64	0.30	0.93	-29.90	1.08	1.69
14.97	SC46.2	460.00	44.00	107.00	0.73	0.32	0.95	-29.90	1.42	1.95
14.58	SC46.3	463.00	42.00	110.00	0.69	0.29	0.90	-30.00	1.05	1.53
14.20	SC48.1	464.00	41.00	117.00	0.69	0.28	1.05	-29.80	0.33	0.48
13.70	SC48.2	463.00	43.00	134.00	0.61	0.26	0.83	-30.10	1.25	2.05
13.20	SC48.3	468.00	40.00	147.00	0.57	0.23	0.80	-29.70	0.80	1.41
12.70	SC48.4	462.00	31.00	167.00	0.48	0.15	0.64	-29.70	1.12	2.34
12.20	SC49	465.00	41.00	122.00	0.58	0.24	0.71	-30.20	1.87	3.23
11.85	SC50.01	469.00	54.00	146.00	0.41	0.22	0.86	-30.10	2.20	5.36
11.52	SC50.02	470.00	50.00	117.00	0.46	0.23	0.64	-29.90	0.07	0.16
11.18	SC50.03	465.00	39.00	118.00	0.61	0.24	0.82	-30.00	0.91	1.49
10.85	SC50.04	466.00	27.00	159.00	0.59	0.16	0.81	-30.10	0.08	0.13
10.51	SC50.05	466.00	41.00	139.00	0.44	0.18	0.51	-30.10	0.08	0.18
10.18	SC50.06	NA	NA	NA	NA	NA	0.56	-30.00	0.07	NA
9.84	SC50.07	NA	NA	NA	NA	NA	0.62	-30.30	0.04	NA
9.51	SC50.08	NA	NA	NA	NA	NA	0.58	-29.60	0.06	NA

9.17	SC50.09	NA	NA	NA	NA	NA	0.53	-29.80	0.04	NA
8.83	SC50.1	NA	NA	NA	NA	NA	0.78	-30.30	0.03	NA
8.50	SC51.1	NA	NA	NA	NA	NA	0.64	-29.60	0.04	NA
8.20	SC51.2	NA	NA	NA	NA	NA	0.68	-29.10	0.04	NA
8.65	Rb_50	NA	NA	NA	NA	NA	0.65	-29.82	0.00	NA
7.90	SC52.1	NA	NA	NA	NA	NA	0.51	-29.80	0.06	NA
7.60	SC52.2	NA	NA	NA	NA	NA	0.64	-29.80	0.04	NA
7.40	Rb_48t	NA	NA	NA	NA	NA	0.79	-29.61	0.00	NA
7.30	SC52.3	NA	NA	NA	NA	NA	0.55	-30.30	0.56	NA
7.00	SC52.4	NA	NA	NA	NA	NA	0.65	-30.40	1.30	NA
6.70	SC52.5	NA	NA	NA	NA	NA	0.95	-30.50	0.04	NA
5.55	Rb_48b	NA	NA	NA	NA	NA	0.58	-29.46	NA	NA
5.30	Rb_46	NA	NA	NA	NA	NA	0.70	-29.72	NA	NA
4.80	Rb_44	NA	NA	NA	NA	NA	0.65	-29.62	NA	NA
4.50	Rb_43	NA	NA	NA	NA	NA	0.15	-27.30	NA	NA
4.35	Rb_42	NA	NA	NA	NA	NA	0.45	-29.60	NA	NA
4.20	Rb_39	NA	NA	NA	NA	NA	0.36	-29.71	NA	NA
3.70	Rb_38	NA	NA	NA	NA	NA	0.29	-29.35	NA	NA
3.20	Rb_36b	NA	NA	NA	NA	NA	0.22	-29.49	NA	NA
2.35	Rb_34	NA	NA	NA	NA	NA	0.20	-29.11	NA	NA
1.65	Rb_32	NA	NA	NA	NA	NA	0.20	-29.18	NA	NA
0.80	Rb_28	NA	NA	NA	NA	NA	0.11	-28.20	NA	NA
0.20	Rb_26	NA	NA	NA	NA	NA	0.23	-28.89	NA	NA
-1.35	Rb_19	NA	NA	NA	NA	NA	0.22	-29.53	NA	NA
-1.80	Rb_18	NA	NA	NA	NA	NA	0.18	-28.72	NA	NA
-2.50	Rb_17	NA	NA	NA	NA	NA	0.16	-28.93	NA	NA
-4.00	Rb_14	NA	NA	NA	NA	NA	0.19	-28.50	NA	NA
-4.40	Rb_13	NA	NA	NA	NA	NA	0.16	-28.10	NA	NA
-4.90	Rb_12	NA	NA	NA	NA	NA	0.12	-28.60	NA	NA
-5.40	Rb_11t	NA	NA	NA	NA	NA	0.10	-27.50	NA	NA
-5.80	Rb_11b	NA	NA	NA	NA	NA	0.08	-27.80	NA	NA
-6.50	Rb_09	NA	NA	NA	NA	NA	0.20	-28.50	NA	NA
-7.00	Rb_07	NA	NA	NA	NA	NA	0.15	-28.20	NA	NA
-7.20	Rb_06	NA	NA	NA	NA	NA	0.11	-27.70	NA	NA
-8.10	Rb_04	NA	NA	NA	NA	NA	0.20	-28.10	NA	NA
-9.40	Rb_01	NA	NA	NA	NA	NA	1.96	-27.70	NA	NA

Re-Os data table TS.4

Sample	Re ppb	± 2s	Total Os ppt	± 2s	$^{187}Re/^{188}Os$	± 2s	$^{187}/^{188}Os$	± 2s	rho	^{187}Re ppb	± 2s	% Re blank	%^{187}Os blk	%^{188}Os blk	initial $^{187}/_{188}$ @ 186.5 Ma
SC37.1	9.95	0.01	504.5	2.2	117.8	0.3	1.97	0.01	0.637	6.25	0.003	2.17	0.01	0.05	1.60
SC39.2	17.00	0.10	322.3	1.8	379.8	2.4	3.92	0.02	0.318	10.69	0.030	1.23	0.01	0.10	2.73
SC40C	63.36	0.02	240.8	1.0	1736.8	3.7	2.96	0.01	0.709	39.83	0.007	0.33	0.01	0.12	-2.44
SC42.1	23.77	0.02	281.8	1.3	596.3	1.3	3.71	0.01	0.666	14.94	0.005	0.83	0.01	0.11	1.85
SC42.2	71.59	0.03	552.8	4.7	870.7	6.2	3.16	0.04	0.641	45.00	0.010	0.28	0.004	0.05	0.45
SC44.2	21.91	0.01	375.2	1.6	408.4	0.8	3.59	0.01	0.687	13.77	0.004	0.98	0.005	0.11	2.32
SC46.1	19.90	0.01	325.1	2.3	431.7	2.1	3.68	0.03	0.639	12.51	0.004	1.00	0.01	0.09	2.34
SC46.3	31.50	0.01	346.9	1.8	676.2	1.7	4.31	0.02	0.676	19.80	0.004	0.64	0.01	0.09	2.20
SC48.3	24.05	0.01	309.6	1.7	541.1	1.7	3.54	0.02	0.653	15.11	0.003	0.93	0.01	0.11	1.86
SC50.3	17.64	0.02	292.8	2.1	402.3	2.2	3.09	0.03	0.644	11.09	0.005	1.28	0.01	0.11	1.83
SC50.5	8.92	0.01	256.8	1.4	227.5	0.8	2.88	0.01	0.663	5.61	0.002	2.42	0.01	0.12	2.17

10. References

1. LaMaskin, T. A., Dorsey, R. J. & Vervoort, J. D. Tectonic Controls on Mudrock Geochemisry, Mesozoic Rocks of Eastern Oregon and Western Idaho, U.S.A.: Implications for Cordilleran Tectonics. *J. Sediment. Res.* **78**, 765–783 (2008).
2. LaMaskin, T. A., Vervoort, J. D., Dorsey, R. J. & Wright, J. E. Early Mesozoic paleogeography and tectonic evolution of the western United States: Insights from detrital zircon U-Pb geochronology, Blue Mountains Province, northeastern Oregon. *Bull. Geol. Soc. Am.* **123**, 1939–1965 (2011).
3. Dorsey, R. J. & LaMaskin, T. A. Stratigraphic record of Triassic-Jurassic collisional tectonics in the Blue Mountains province, northern Oregon. *Am. J. Sci.* **307**, 1167–1193 (2007).
4. Smith, P. L., Tipper, H. W., Taylor, D. G. & Guex, J. An ammonite zonation for the Lower Jurassic of Canada and the United States: the Pliensbachian. *Can. J. Earth Sci.* **25**, 1503–1523 (1988).
5. Caruthers, A. H. *et al.* Pliensbachian-Toarcian (Early Jurassic) ammonoids from the Luning Embayment, west-central Nevada, U.S.A. *Bull. Am. Paleontol.* **393**, 1–83 (2018).
6. Gézcy, B. & Meister, C. Les ammonites du Domérien de la montagne du Bakony (Hongrie). *Rev. Paléobiologie* **17**, 69–161 (1998).
7. Meister, C. & Blau, J. Pliensbachian ammonites from the Central Apennines, Italy (Acquasparta section) – a revision of Fischer's collection and new data. *Neues Jahrb. für Geol. und Paläontologie* **273**, 253–275 (2014).
8. Meister, C., Dommergues, J.-L., Dommergues, C., Lachkar, N. & El Hariri, K. Les ammonites du Pliensbachien du Jebel Bou Rharraf (Haut Atlas oriental, Maroc). *Geobios* **44**, 1–117 (2011).
9. Meister, C., Schirolli, P. & Dommergues, J. L. Early Jurassic (Sinemurian to basal Toarcian) ammonites of the Brescian Prealps (Southern Alps, Italy). *Riv. Ital. di Paleontol. e Stratigr.* **123**, 79–148 (2017).
10. Hillebrandt, A. V. Ammoniten aus dem Pliensbachium (Carixium und Domerium) von Südamerika. *Rev. Paleobiol.* **25**, 1–403 (2006).
11. Smith, P. L. & Tipper, H. W. Pliensbachian (Lower Jurassic) ammonites of the Queen Charlotte Islands, British Columbia. *Bull. Am. Paleontol.* **108**, 1–122 (1996).
12. Caruthers, A. H. & Smith, P. L. Pliensbachian ammonoids from the Talkeetna Mountains (Peninsular Terrane) of Southern Alaska. *Rev. Paléobiologie* **11**, 365–378 (2012).
13. Johannson, G. G., Smith, P. L. & Gordey, S. P. Early Jurassic evolution of the northern Stikinian arc: evidence from the Laberge Group, northwestern British Columbia. *Can. J. Earth Sci.* **34**, 1030–1057 (1997).
14. Meister, C. Les ammonites du domérien des Causses (France). Analyses paléontologiques et stratigraphiques. *Cah. Paléontologie* 98 (1989).
15. Da Rocha, R. B. *et al.* Base of the Toarcian Stage of the Lower Jurassic defined by the Global Boundary Stratotype Section and Point (GSSP) at the Peniche section (Portugal). *Episodes* **39**, 460–481 (2016).
16. Fauré, P., Améras, Y., Sekatni, N. & Zargouni, F. Le Pliensbachien de Jebel Zaghouan (Tunisie). Nouvelles données fauniques. Implications biostratigraphiques et pléobiogéographiques. *Geodiversitas* **29**, 473–505 (2007).
17. Montero-Serrano, J. C. *et al.* Continental weathering and redox conditions during the early Toarcian Oceanic Anoxic Event in the northwestern Tethys: Insight from the Posidonia

Shale section in the Swiss Jura Mountains. *Palaeogeogr. Palaeoclimatol. Palaeoecol.* **429**, 83–99 (2015).

18. Fantasia, A., Föllmi, K. B., Adatte, T., Spangenberg, J. . & Mattioli, E. Expression of the Toarcian Oceanic Anoxic Event: New insights from a Swiss transect. *Sedimentology* **66**, 262–284 (2019).
19. Fantasia, A., Föllmi, K. B., Adatte, T., Spangenberg, J. . & Montero-Serrano, J.-C. The Early Toarcian oceanic anoxic event: Paleoenvironmental and paleoclimatic change across the Alpine Tethys (Switzerland). *Glob. Planet. Change* **162**, 53–68 (2018).
20. Schootbrugge, B. Van De *et al.* Early Jurassic climate change and the radiation of organic- walled phytoplankton in the Tethys Ocean. *Paleobiology* **31**, 73–97 (2005).
21. Mailliot, S. *et al.* Late Pliensbachian–Early Toarcian (Early Jurassic) environmental changes in an epicontinental basin of NW Europe (Causses area, central France): A micropaleontological and geochemical approach. *Palaeogeogr. Palaeoclimatol. Palaeoecol.* **273**, 346–364 (2009).
22. Jaffey, A. H., Flynn, K. F., Glendenin, L. E., Bentley, W. C. & Essling, A. M. Precision measurement of half-lives and specific activities of U235 and U238. *Phys. Rev. C* **4**, 1889–1906 (1971).
